# Classification of intestinal T‐cell receptor repertoires using machine learning methods can identify patients with coeliac disease regardless of dietary gluten status

**DOI:** 10.1002/path.5592

**Published:** 2021-01-06

**Authors:** Andrew D Foers, M Saad Shoukat, Oliver E Welsh, Killian Donovan, Russell Petry, Shelley C Evans, Michael EB FitzPatrick, Nadine Collins, Paul Klenerman, Anna Fowler, Elizabeth J Soilleux

**Affiliations:** ^1^ Department of Pathology University of Cambridge Cambridge UK; ^2^ Centre for Mathematical Sciences University of Cambridge Cambridge UK; ^3^ Oxford University Medical School Oxford UK; ^4^ Translational Gastroenterology Unit, Nuffield Department of Medicine University of Oxford Oxford UK; ^5^ Department of Molecular Pathology Royal Surrey NHS Foundation Trust Guildford UK; ^6^ Peter Medawar Building for Pathogen Research University of Oxford Oxford UK; ^7^ Department of Health Data Science, Institute of Population Health University of Liverpool Liverpool UK; ^8^ Nuffield Division of Clinical Laboratory Sciences, Radcliffe Department of Medicine University of Oxford Oxford UK

**Keywords:** coeliac disease, gluten, T‐lymphocyte, T‐cell receptor repertoire, machine learning, TRG, TRD, clustering, duodenum

## Abstract

In coeliac disease (CeD), immune‐mediated small intestinal damage is precipitated by gluten, leading to variable symptoms and complications, occasionally including aggressive T‐cell lymphoma. Diagnosis, based primarily on histopathological examination of duodenal biopsies, is confounded by poor concordance between pathologists and minimal histological abnormality if insufficient gluten is consumed. CeD pathogenesis involves both CD4^+^ T‐cell‐mediated gluten recognition and CD8^+^ and γδ T‐cell‐mediated inflammation, with a previous study demonstrating a permanent change in γδ T‐cell populations in CeD. We leveraged this understanding and explored the diagnostic utility of bulk T‐cell receptor (TCR) sequencing in assessing duodenal biopsies in CeD. Genomic DNA extracted from duodenal biopsies underwent sequencing for TCR‐δ (TRD) (CeD, *n* = 11; non‐CeD, *n* = 11) and TCR‐γ (TRG) (CeD, *n* = 33; non‐CeD, *n* = 21). We developed a novel machine learning‐based analysis of the TCR repertoire, clustering samples by diagnosis. Leave‐one‐out cross‐validation (LOOCV) was performed to validate the classification algorithm. Using TRD repertoire, 100% (22/22) of duodenal biopsies were correctly classified, with a LOOCV accuracy of 91%. Using TCR‐γ (TRG) repertoire, 94.4% (51/54) of duodenal biopsies were correctly classified, with LOOCV of 87%. Duodenal biopsy TRG repertoire analysis permitted accurate classification of biopsies from patients with CeD following a strict gluten‐free diet for at least 6 months, who would be misclassified by current tests. This result reflects permanent changes to the duodenal γδ TCR repertoire in CeD, even in the absence of gluten consumption. Our method could complement or replace histopathological diagnosis in CeD and might have particular clinical utility in the diagnostic testing of patients unable to tolerate dietary gluten, and for assessing duodenal biopsies with equivocal features. This approach is generalisable to any TCR/BCR locus and any sequencing platform, with potential to predict diagnosis or prognosis in conditions mediated or modulated by the adaptive immune response. © 2020 The Authors. *The Journal of Pathology* published by John Wiley & Sons, Ltd. on behalf of The Pathological Society of Great Britain and Ireland.

## Introduction

Coeliac disease (CeD) is a gluten‐sensitive enteropathy that develops in genetically susceptible individuals who are exposed to cereal gluten proteins, found in wheat, rye, and barley. Much of the genetic susceptibility is contributed by possession of the MHC class II molecules HLA‐DQ2 and HLA‐DQ8. Proteins encoded by these genes bind gluten peptides, particularly those peptides post‐translationally modified by tissue transglutaminase (tTG). Recognition of MHC‐bound gluten peptide antigens by CD4^+^ T‐lymphocytes induces inflammation, which damages the small intestine, leading to malabsorption, which, in turn, causes many of the symptoms of CeD [1]. The key histopathological changes identifiable in CeD duodenum are mainly consequences of the T‐cell response and include villous atrophy, crypt hyperplasia, and increased numbers of mucosal lymphocytes, which are a mixture of γδ and CD8^+^ αβ T‐cells [1, 2, 3]. While most of the inflammation disappears on a gluten‐free diet (GFD), numbers of intraepithelial γδ T‐cells remain elevated [2, 3, 4, 5, 6, 7, 8]. A recent study elegantly demonstrated that not only do the γδ T‐cell numbers increase permanently, once tolerance to gluten is lost, but that there is also an irreversible alteration of the functional subtypes of γδ T‐cells present in the duodenum. They demonstrated depletion of naturally occurring, innate‐like Vγ4^+^/Vδ1^+^ intraepithelial lymphocytes (IELs) with specificity for the butyrophilin‐like (BTNL) molecules BTNL3/BTNL8, expressed in the duodenum. In tandem, they observed expansion of gluten‐sensitive, interferon‐γ‐producing Vδ1^+^ IELs bearing T‐cell receptors (TCRs) with a shared non‐germline‐encoded motif that failed to recognise BTNL3/BTNL8 and were phenotypically more akin to adaptive T‐cells [4].

CeD treatment is a strict gluten‐free diet to avoid complications of malabsorption (vitamin/mineral deficiency, weight loss, anaemia, infertility, osteoporosis), linked immune phenomena (dermatitis herpetiformis, microscopic colitis), and, rarely, enteropathy‐associated T‐cell lymphoma. Clinical presentations of CeD are variable, and include non‐specific gastrointestinal symptoms (abdominal pain, bloating, diarrhoea), fatigue, and cognitive difficulty [1, 9, 10]. The estimated prevalence of CeD in the UK and US population is 1% [10, 11] and rising [12, 13]. Screening studies suggest that up to 90% of cases remain undiagnosed [11]. An increasing proportion of the population follows a self‐imposed gluten‐free diet (GFD) without a CeD diagnosis [14].

Adult CeD testing strategies comprise serology for anti‐tissue transglutaminase (tTG) and anti‐endomysial antibody (EMA), and histopathological examination of duodenal endoscopic biopsies, the latter remaining the diagnostic ‘gold standard’ [10, 11]. Biopsy examination by a pathologist is unavoidably subjective, with poor inter‐observer concordance [15], variable concordance with serology [16], and a high rate of ‘equivocal’ biopsies [2]. Both serology and endoscopic biopsy require patients to eat appreciable amounts of gluten for 6 weeks prior to testing to avoid false‐negative or equivocal results [17], meaning that many gluten‐sensitive patients choose not to seek testing, due to the unpleasant symptoms that follow gluten ingestion. There is an unmet need for a more robust and objective test to diagnose CeD in patients, irrespective of gluten intake, particularly for patients with severe gluten‐induced symptoms.

TCRs determine the antigen(s) a T‐cell can bind and respond to and are heterodimers of TCR‐αβ and TCR‐γδ type. A randomly selected and recombined variable (*V*) and joining (*J*) segment encode the antigen binding region of TCR α‐ and γ‐chains (encoded by the TRA and TRG genes, respectively), while TCR β‐ and δ‐chains (encoded by the TRB and TRD genes, respectively) are encoded by *V*, *J*, and diversity (*D*) regions [18]. In addition to this somatic recombination, template‐independent nucleotide insertion and deletion occur, meaning that the small set of TCR genes can theoretically create 10^15^–10^20^ unique TCR clonotypes. The most variable part of the TCR, encoded by the V(D)J junction, known as the complementarity‐determining region 3 (CDR3) is critical in determining antigen specificity [19] and can be used as a genetic ‘barcode’ to detect, track, and analyse T‐cells. The TCR repertoire (TCRR) refers to the range of different TCRs expressed and is shaped by previously encountered antigens [20, 21].

Clinically, TCRs are only assessed when PCR and fragment analysis of TCR sequences are used to assess clonal status in suspected T‐cell lymphoma [22]. Bulk sequencing of TCRRs is also an important research tool [18], capturing the V(D)J regions in large numbers of T‐cells. Although this produces large datasets, there are few machine learning algorithms for diagnosing immunological conditions from TCRRs [21], with none in clinical use. Furthermore, many studies use only a fraction of the total information in a TCRR dataset, which comprises multiple closely related, but distinct, TCR sequences. Previous studies of the TCRR of CeD patients have focused on identifying one or a few previously identified TCR sequences, identifying shared motifs between different individuals [23], quantifying sequence diversity in a sample using Shannon diversity or on assessing the magnitude of clonal expansions [4, 23, 24, 25, 26, 27, 28, 29, 30, 31, 32, 33]. Very few studies have undertaken a comparison of patient groups by means of holistic analysis of TCRR sequence data [34, 35], due to a lack of bioinformatic tools for doing so. Here, we undertake bioinformatic analysis of the entire TCRR, derived from a duodenal biopsy, in order to classify patients according to their gluten sensitivity status. We show that our approach is successful regardless of whether or not the patient's diet contained gluten prior to biopsy.

## Materials and methods

### Ethical approval, patient samples, and DNA extraction

Fully anonymised, formalin‐fixed, paraffin‐embedded (FFPE) duodenal biopsy samples surplus to diagnostic requirements were obtained from the Oxford Radcliffe Biobank via the Oxford Centre for Histopathology Research, or from the Human Tissue Research Biobank, Cambridge University Hospitals NHS Foundation Trust, with full ethical approval (IRAS: 162057). Specific informed consent from individual patients was not required for entry into this study, as all the samples used were (i) surplus to diagnostic requirements and (ii) fully anonymised to the research team. Patient details and criteria for inclusion are included in Table 1 and supplementary material, Table S1. We obtained duodenal biopsies from patients with active CeD [at least Marsh 3b (*n* = 43); Marsh 2 (*n* = 1) [36]] and from non‐CeD individuals (*n* = 32) with no clinical or histological suspicion of CeD (undergoing upper gastrointestinal endoscopy for clinical indications of reflux, dyspepsia, and gastritis). We also obtained duodenal biopsies from CeD patients, with a previous biopsy showing at least Marsh 3b features, who had been on a strict GFD for at least 6 months with normal duodenal biopsy histopathology (*n* = 4). DNA was extracted from ten FFPE scrolls cut at 5 μm per case using the QIAamp DNA FFPE tissue kit (Qiagen, Manchester, UK), according to the manufacturer's instructions.

**Table 1:** Patient demographics and clinical parameters.

TRD (DNA)			
	Coeliac (*n =* 11)	Normal (*n =* 11)	*P* value
Age, mean (± SD)	29.2 (20.8)	53.8 (13.3)	0.002
Sex (M : F)	4 : 7	3 : 8	1.00
HLA‐DQ2 and/or HLA‐DQ8	11 (100%)	3 (27%)	0.001
Anti‐TTG (>laboratory ULN)	11 (100%)	0 (0%)	<0.0001
Anti‐EMA (>laboratory ULN)	11 (100%)	0 (0%)	<0.0001
Marsh grade (3;2;1;0)	10;1;0;0	0;0;1;10	<0.0001*

TRG (DNA)			
	Coeliac (*n =* 33)	Normal (*n =* 21)	*P* value
Age, mean (± SD)	16.0 (9.2)	54.8 (17.9)	<0.0001
Sex (M : F)	8 : 25	12 : 9	0.021
HLA‐DQ2 and/or HLA‐DQ8	32 (100%)^†^	11 (52%)	<0.0001
Anti‐TTG (>laboratory ULN)	33 (100%)	0 (0%)	0.140
Anti‐EMA (>laboratory ULN)	28 (85%)	0 (0%)	<0.0001
Marsh grade (3;2;1;0)	33;0;0;0	0;0;0;21	<0.0001*

Gluten‐free diet longitudinal patients (*n* = 4)			
	Baseline	6 months GFD	*P* value
Age, mean (± SD)	39.5 (13.6)	Same patients	–
Sex (M : F)	0 : 4	Same patients	–
Anti‐TTG (>laboratory ULN)	4 (100%)	0 (0%)	–
Anti‐EMA (>laboratory ULN)	3 (75%)	0 (0%)	–
Marsh grade (3;2;1;0)	4;0;0;0	0;0;0;4	–

Age and Marsh grade analysed with Student's *t*‐test. Sex, HLA status, anti‐TTG, and anti‐EMA analysed with Fisher's exact test.

ULN, upper limit of normal.

* Calculated from average of Marsh grades.

^†^ HLA typing inconclusive for patient C31.

### TCR repertoire sequencing

Bulk amplification of T‐cell receptor repertoires was undertaken with the Biomed‐2 kit (Invivoscribe, Martinsried, Bavaria, Germany) for TCRδ (TRD), using 150 ng DNA input. An equal amount of all purified amplicons was pooled into a library of 4 nm, denatured, diluted, loaded onto a MiSeq (Illumina, San Diego, CA, USA), and subjected to a MiSeq run (Illumina; v3, 2 × 300 cycles). For TCRγ (TRG), the LymphoTrack kit (Invivoscribe) was used following the manufacturer's instructions, with 200 ng of duodenal biopsy DNA as a template. Primers in the LymphoTrack (Invivoscribe) assays are designed with Illumina adapters. Subsequently, each amplicon was purified by AMPure XP beads (Beckman Coulter, Brea, CA, USA) followed by quantification using a 2100 Bioanalyzer (Agilent, Santa Clara, CA, USA). Samples were pooled in an equimolar fashion and sequenced on an Ion PGM (Thermo Fisher Scientific, Loughborough, UK).

### Bioinformatic analysis

FastX [37] was used to remove low‐quality sequences. Low‐quality sequences were defined as individual sequences with more than 10% of nucleotides achieving a quality score below the third quartile (lower 25%). Quartiles were determined using the average nucleotide score for the sequencing output on a per patient basis. Reads were then aligned to the IMGT reference database (http://www.imgt.org) using IMGT/HighV‐Quest (http://www.imgt.org/IMGTindex/IMGTHighV‐QUEST.php [38]*)*, in order to determine V, D, and J usage, to identify the CDR3 region, and to determine whether or not a sequence was functional (supplementary material, Table S2). Nucleotide sequences were translated to amino acids, which are used in all subsequent analysis, and all sequences predicted to be non‐functional were removed at this stage.

### Classification algorithm

**Figure 1:**
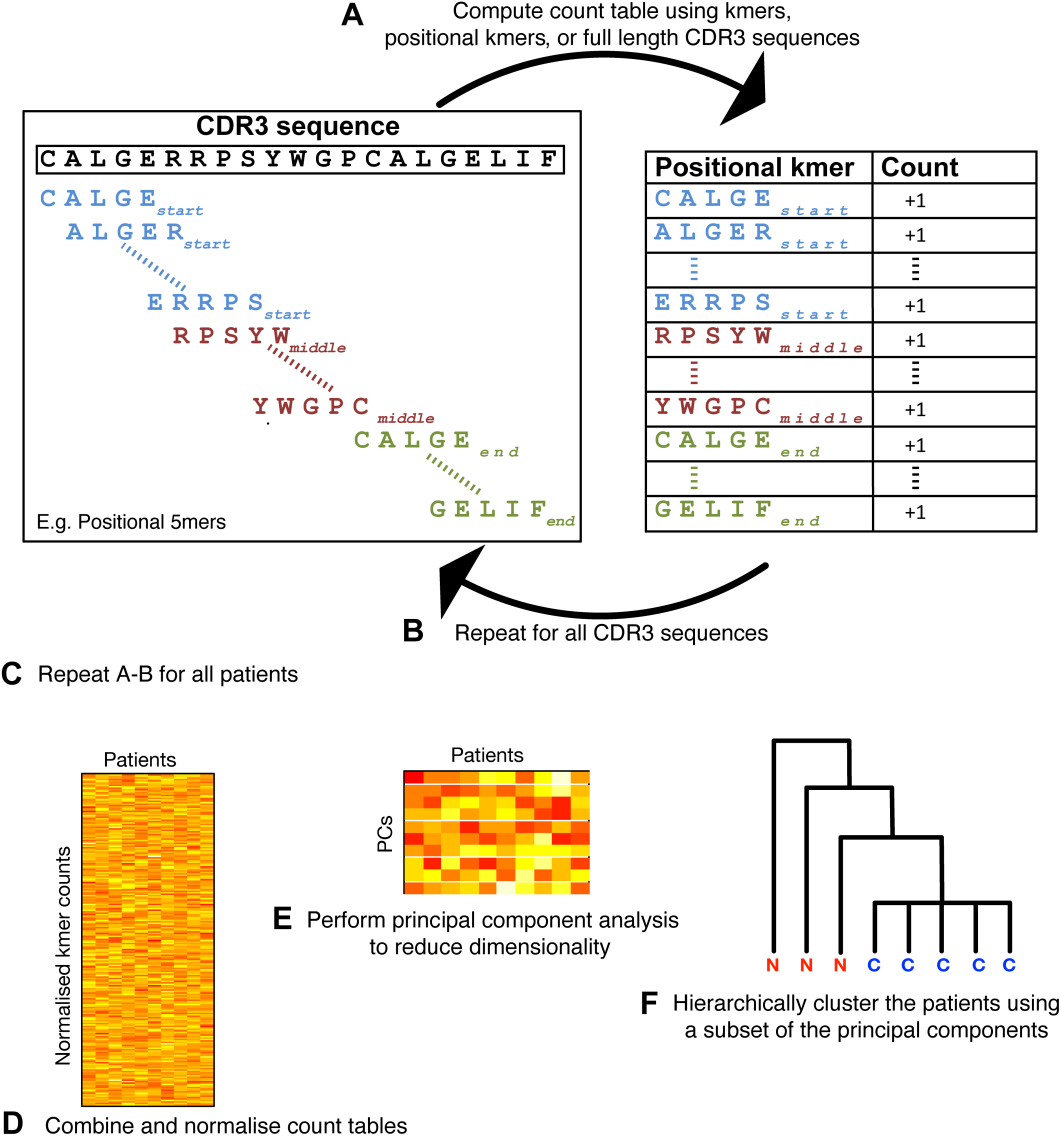
Flow chart of our novel bioinformatic approach. (A) We translate the nucleic acid sequence to amino acid sequences, remove any non‐functional sequences, and identify the most variable of the three hypervariable regions in each TCR, the CDR3 region, using the IMGT database [38]. To take account of similar, but not clonotypically identical TCR sequences, we break the entire CDR3 sequence of each TCR into short overlapping segments, designated *k*mers. We reasoned that the same *k*mer occurring at substantially different positions within the CDR3 is likely to differ in its effect on antigen binding and so tested positionally annotated *k*mers (start/middle/end) in the functional CDR3s. For example, CALGE (start) is regarded as distinct from CALGE (end). (B) We calculate the frequency of each full‐length CDR3, unique *k*mer, and positional *k*mer identified in step A. (C) Steps A and B are repeated for each patient, so that sample classification results using each of these three different input types can be compared. (D) Frequencies are normalised for each sample and combined for all samples into a single frequency matrix. (E) The frequency matrices are particularly high dimensional, due to the very large number of possible positional *k*mers (4.8 × 10^5^ to 1.5 × 10^12^ for 4–9 amino acid *k*mers, respectively). Therefore, principal component analysis (PCA) is used to reduce this dimensionality, while retaining major sources of variation, simplifying downstream computational steps. (F) To classify samples, we apply hierarchical clustering to the dimensionality reduced data set, which iteratively groups together samples. Samples for which the true underlying disease status is known are used to select optimal parameter sets, consisting of the value of *k* and principal components (PCs), as well as input type (full‐length CDR3, non‐positional *k*mers, and positional *k*mers), by means of a machine learning approach (i.e. to train the model). The optimal parameters generate clusters that correspond best with disease state (see supplementary material, Supplementary materials and methods).

A novel cluster‐based classification algorithm for distinguishing between the TCRR of case and control samples was developed and is described in the Results section (Figure 1). The algorithm, implemented in R scripts, is available from Zenodo (http://doi.org/10.5281/zenodo.3964131).

### Assessment of classification performance

During clustering, we partition the tree into two clusters by choosing a merge (node) and defining the cluster created at that merge as a single cluster, then assigning all other samples (leaves) into another single cluster. The two clusters are then labelled either CeD or non‐CeD according to the true classification of the majority of samples in each cluster. Sensitivity is then calculated as the percentage of CeD samples correctly labelled, specificity as the percentage of non‐CeD samples correctly labelled, and (training) accuracy as the percentage of all samples correctly labelled. The optimal partition was chosen as the one with highest accuracy, and in the case of ties, partitions were then ranked based on sensitivity and finally distance between clusters (further details for clustering methodology and parameter optimisation are presented in supplementary material, Supplementary materials and methods).

### Leave‐one‐out cross‐validation

In order to provide a validation of our methodology and illustrate how the algorithm could be used for diagnosis, algorithm parameters were optimised using *n* − 1 samples with one sample removed. The removed sample was then re‐introduced to determine whether it was assigned to the correct group, on the basis of diagnosis. This process was repeated iteratively until each sample had been removed to estimate the testing accuracy of the algorithm (further details are presented in supplementary material, Supplementary materials and methods, together with a more detailed explanation of the statistical methodology used in all analyses).

### Heatmap and hierarchical clustering analysis based on V and J segment usage

V and J segment usage is visualised by heatmap using the pheatmap R package [39]. V and J segment frequency across patients is represented as standard deviations from the mean. Hierarchical clustering was performed on columns using the complete linkage method with Euclidean distance.

## Results

### A novel machine learning algorithm for sample classification

We developed an algorithm capable of diagnosing CeD, regardless of gluten consumption, on the basis of TCRR in duodenal biopsies (Figure 1). Our approach was based on the hypothesis that there are multiple related γδ TCRs with similar specificities, capable of binding gluten and possibly self‐antigens, due to the phenomenon of epitope spreading [40], encoded by closely related TRG or TRD sequences [4, 23], both within a single patient's TCRR and between patient TCRRs with CeD. In brief, TCR sequences are translated into amino acids and the hypervariable part of the TCR sequence, complementarity determining region 3 (CDR3), is broken into overlapping *k*mers (sequences of length *k*). We produce a set of *k*mers that is positionally annotated, by which third of the CDR3 sequence they derive from (start/middle/end), and a set that lacks this annotation. We compile a very large (high dimensionality) matrix containing the frequency of each *k*mer in each patient sample. We reduce the dimensionality of the matrix by principal component analysis (PCA) and cluster the samples using all 1023 possible combinations of principal components (PCs) 1–10. We then determine which of these PC combinations has the highest sample classification accuracy. Of these PC combinations with high sample classification accuracy, we then select the PC combination that gives the greatest separation between diagnostic groups. We tested our approach on both the positionally annotated and the non‐positionally annotated sets of *k*mers, testing parameters, as described above, and determined whether or not positionally annotating the *k*mers improved samples' classification. Thus, in this machine learning approach, the modifiable parameters are (i) *k*mer length, (ii) whether or not *k*mers are positionally annotated, and (iii) the exact PC combination used for sample classification.

### TCR delta repertoire analysis of FFPE duodenal biopsy DNA can determine gluten sensitivity status

**Figure 2:**
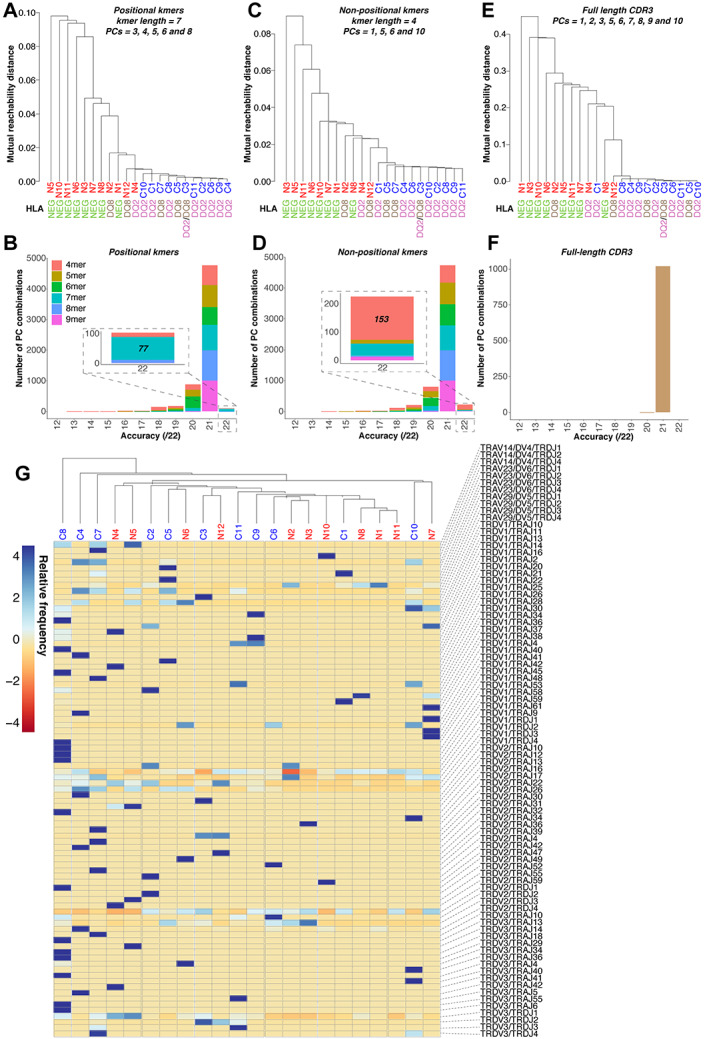
Application of our algorithm to TRD sequence data obtained from formalin‐fixed, paraffin‐embedded duodenal biopsies of coeliac disease patients (*n* = 11) and non‐coeliac disease controls (*n* = 11). Diagnostic classification accuracy was optimised using all possible input types (positional *k*mers, non‐positional *k*mers, and full‐length CDR3 sequences) and PC combinations. (A, B) With positional annotation, a *k*mer length of 7 achieved greatest accuracy, with 77/1023 PC combinations giving 100% accuracy (supplementary material, Table S4). Of the 77 PC combinations giving 100% accuracy, PCs 3, 4, 5, 6, and 8 gave the greatest separation between diagnostic groups, with the greatest vertical distance between branches on the cluster plot (known technically as the mutual reachability distance). HLA type (DQ2 and/or DQ8 or other) does not explain the classification, with non‐CeD samples from DQ2 or DQ8 positive subjects clustering on the basis of disease (see also Figure 2C,E). (C, D) Without positional annotation, a *k*mer length of 4 was optimal and 153/1023 PC combinations gave 100% accuracy (supplementary material, Table S3). Of the 153 PC combinations giving 100% accuracy, PCs 1, 5, 6, and 10 gave the greatest separation between diagnostic groups, with the greatest vertical distance between branches on the cluster plot. The high classification accuracy across a broad range of parameters indicates the robustness of this approach. (E, F) Using full‐length CDR3 sequences, no PC combinations gave 100% accuracy, although 1021 PC combinations permitted 21/22 (95.5%) samples to be classified correctly (supplementary material, Table S5). (G) Hierarchical clustering on the basis of combinations of V–J segments usage in the sequence data could not classify patient samples by diagnosis. Patient details and inclusion criteria for the TRD sequencing cohort may be found in Table 1 and supplementary material, Table S1. Sequence data parameters are summarised in supplementary material, Table S2. Further validation of these results is included in supplementary material, Figures S1–S3 and Tables S6–S8.

We applied our algorithm to TRD CDR3 sequences, from DNA extracted from CeD patient duodenal biopsies (*n* = 11) and non‐CeD controls (*n* = 11) (supplementary material, Table S1). Diagnostic accuracy was optimised (Figure 2) by selecting (i) *k*mer length and (ii) *k*mer type (positionally annotated versus not, as defined in Figure 1), and (iii) PCs. Non‐positionally annotated 4mers gave optimal sample separation by diagnosis, with high accuracy across a broad range of PC combinations, with 14.96% PC combinations (153/1023) giving 100% training accuracy (Figure 2C,D and supplementary material, Table S3). With positional annotation, *k*mer length of 7 was optimal, with 7.53% combinations (77/1023) giving 100% training accuracy (Figure 2A,B and supplementary material, Table S4). Of 153/1023 PC combinations giving 100% accuracy with non‐positionally annotated 4mers, the PC combination of 1, 5, 6, and 10 gave greatest separation between diagnostic groups, with greatest vertical distance between cluster plot branches (Figure 2C). From Figure 2B,D, it can be appreciated that a wide range of *k*mer lengths and PC combinations also gave good sample classification, indicating the robustness of our approach. To validate our optimised classification algorithm using non‐positional 4mers, we implemented a leave‐one‐out cross‐validation (LOOCV) approach (supplementary material, Figure S3). Ten of 11 CeD and 10/11 non‐CeD patients clustered correctly, giving a testing accuracy, sensitivity, and specificity of 91%. We excluded the possibility that HLA type or other properties of the TRD data might be confounding our classification methodology (supplementary material, Figures S1 and S2 and Tables S4 and S5–S8; Figure 2A,C).

### TCR gamma repertoire analysis of FFPE duodenal biopsy DNA can determine gluten sensitivity status

**Figure 3:**
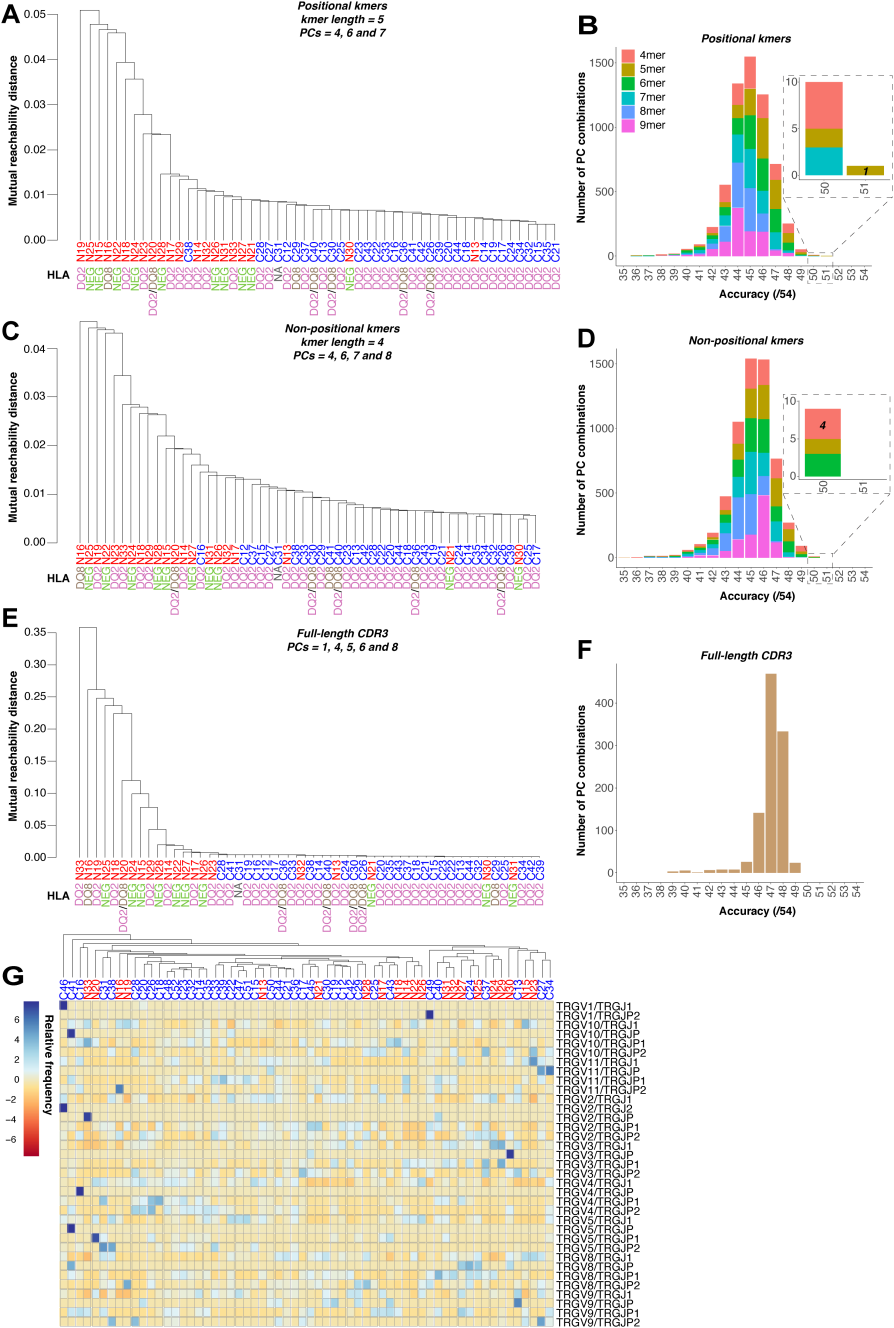
Application of our algorithm to TRG sequence data obtained from formalin‐fixed, paraffin‐embedded duodenal biopsies of coeliac disease patients (*n* = 33) and non‐coeliac disease controls (*n* = 21). Diagnostic classification accuracy was optimised using all possible input types (positional *k*mers, non‐positional *k*mers, and full‐length CD3 sequences) and PC combinations. (A, B) With positional annotation, a *k*mer length of 5 achieved greatest accuracy, with one PC combination (PCs 4, 6, and 7) classifying samples with 94.4% accuracy (supplementary material, Table S9). (C, D) Without positional annotation, a *k*mer length of 4 was optimal, with four PC combinations giving 92.6% accuracy (supplementary material, Table S10). HLA type (DQ2 and/or DQ8 or neither) did not explain the classification, with non‐CeD samples from DQ2 or DQ8 positive subjects clustering on the basis of disease (see also Figure 3A). (E, F) Using full‐length CDR3 sequences, 24 PC combinations gave 90.7% accuracy (supplementary material, Table S11). (G) To exclude the possibility that other properties of the data might be confounding our classification methodology, we undertook analysis of V and J segment usage, shown as a heatmap, and showed that hierarchical clustering on the basis of combinations of V–J segments usage in the sequence data could not cluster patient samples by diagnosis. Patient details and inclusion criteria for the TRG sequencing cohort may be found in Table 1 and supplementary material, Table S1. Further validation of these results is included in supplementary material, Figures S4–S6 and Tables S12–S15.

Our algorithm also performed well in classifying patients' CeD status using TRG repertoires derived from DNA extracted from FFPE duodenal biopsies. In a second, larger patient cohort (*n* = 54), TRG CDR3 sequences were broken into positional 5mers and the PC combination of 4, 6, and 7 was best able to separate the patient cohorts, with 32/33 (97.0% training sensitivity) CeD and 19/21 (90.5% training specificity) non‐CeD samples correctly classified, giving a training accuracy of 51/54 (94.4%) (Figure 3A,B and supplementary material, Table S9). Figure 3B,D shows that a wide range of *k*mer lengths and PC combinations also gave good sample classification, indicating the broad applicability of our approach to this analysis (supplementary material, Table S9). For TRG, in contrast to TRD, positionally annotated *k*mers outperformed non‐positionally annotated *k*mers (Figure 3C,D and supplementary material, Table S10). Neither full‐length CDR3 (Figure 3E,F and supplementary material, Table S11) nor V/D/J segment usage was able to accurately separate CeD from non‐CeD samples (Figure 3G and supplementary material, Figure S4D,E). We also excluded the possibility that HLA type or other properties of the TRG data might be confounding our classification methodology (supplementary material, Figures S4 and S5 and Tables S12–S15; Figure 3A,C).

**Figure 4:**
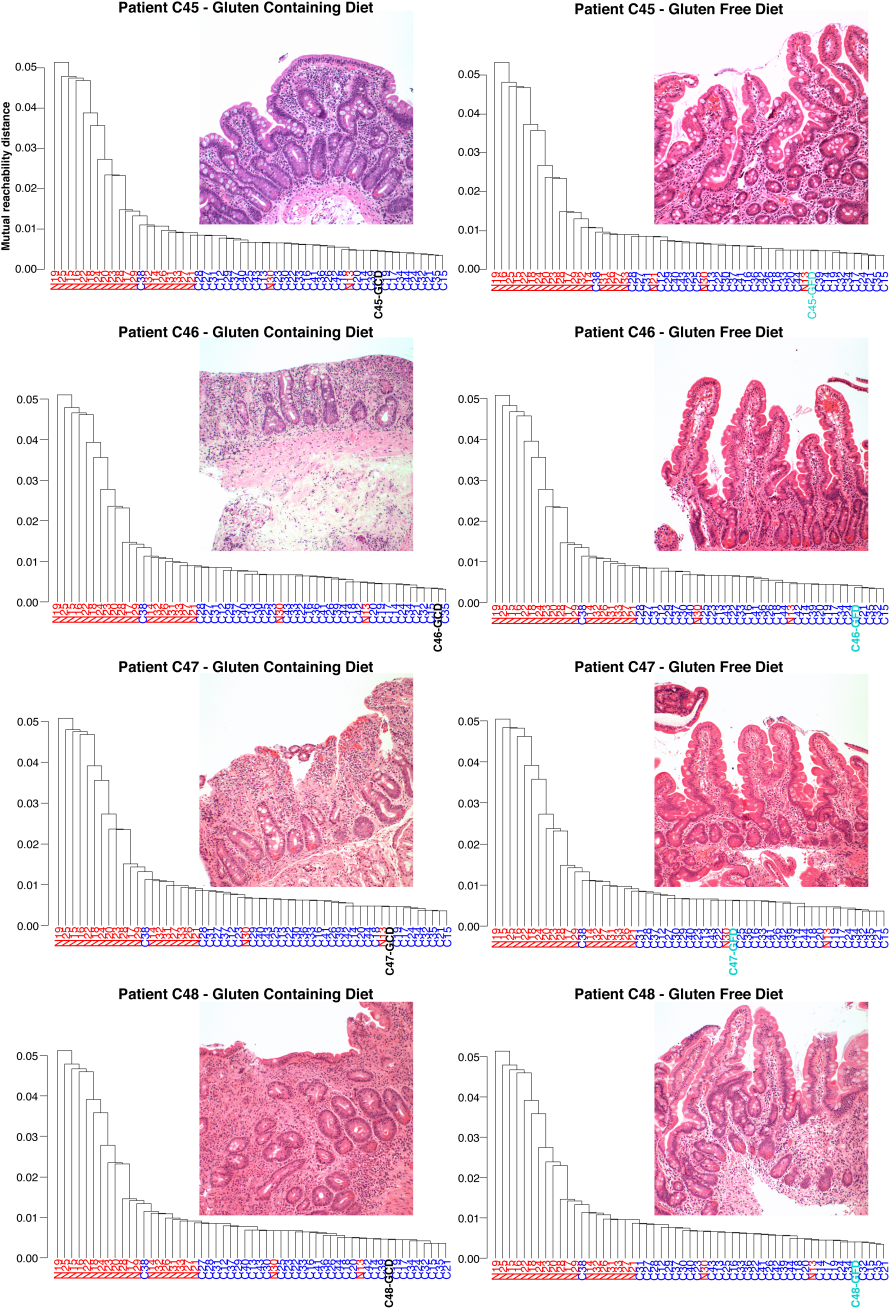
Applicability of TRG analysis to patient samples on a gluten‐free diet. Four additional formalin‐fixed, paraffin‐embedded duodenal biopsy samples from patients on a gluten‐containing diet (i.e. at the time of initial diagnosis of coeliac disease) were obtained. Histology (haematoxylin and eosin stain) of each sample is shown, all with severe features of coeliac disease (at least Marsh grade 3a). TRG sequence data obtained from the biopsy samples shown were added one by one into the cohort and analysed by means of our algorithm. Each patient sample correctly clustered with the coeliac samples. An additional duodenal biopsy sample was taken from each patient following at least 6 months on a GFD. Histology (haematoxylin and eosin stain) of each sample is shown and all would be classified as histologically normal. TRG sequence data obtained from biopsy samples were added one by one into the cohort and analysed by means of our algorithm. Again, each patient sample correctly clustered with the coeliac samples.

Implementing a LOOCV approach to validate our preliminary findings for TRG, using positional 5mers, 29/33 CeD and 18/21 non‐CeD samples were correctly classified, giving a testing accuracy of 87%, a sensitivity of 88%, and a specificity of 86% (supplementary material, Figure S6).

### Gluten intake is not required for correct classification of patient gluten sensitivity status

Finally, we assessed whether our method could classify patients diagnosed with CeD who were adhering to a GFD. Duodenal biopsies were obtained from an additional four patients, each comprising one sample at initial CeD diagnosis, when the patient was on a gluten‐containing diet [with changes of at least Marsh–Oberhuber [36] grade 3b (Figure 4)], and a second sample following at least 6 months on a strict GFD that displayed normal histology (Figure 4). We introduced the additional patient samples that were taken at initial CeD diagnosis into the 54‐TRG cohort one at a time, using our previously optimised parameters, and all samples clustered with the CeD samples. Remarkably, all four GFD samples, when introduced individually into the cohort, also clustered with the CeD samples (Figure 4). These data indicate that our algorithm is capable of identifying patients with CeD even in the absence of gluten ingestion.

## Discussion

Our novel machine learning approach distinguished samples from patients with and without CeD, when applied to TCRR (TRD and TRG) from duodenal DNA from two independent cohorts, despite using a relatively degraded FFPE‐derived DNA template, most likely leading to loss of a proportion of the TCR sequences in each sample. The sequencing approaches that we selected (Lymphotrack™/Biomed‐2, Invivoscribe, with Illumina sequencing) are amenable to FFPE‐derived DNA and are used clinically in lymphoma/leukaemia diagnostics. Thus, our approach to CeD diagnosis could easily be incorporated into current pathology department workflows. Following a larger validation study of diagnostic accuracy [41], this algorithm could have the potential for use in the diagnosis of CeD in cases where current diagnostic techniques do not perform well and where pathologists struggle to agree on histological findings, such as those with an isolated increase in intraepithelial lymphocytes without villous atrophy, those with seronegative villous atrophy, and those CeD patients on a GFD with normal histology, who are likely to be misclassified by current serological and histopathological testing [2, 15, 16, 17, 42]. Although only a small number of patients on GFDs were analysed here, our approach shows promise in eradicating the requirement for patients to ingest gluten for 2–6 weeks prior to testing [43, 44], increasing test acceptability to patients.

The current definition of CeD is hampered by imperfect, and sometimes discordant, tests for the condition. Our algorithm has the exciting potential to provide a better definition of CeD based on similarities between patients' TCRRs. Further refinement of diagnostic classification might be achieved by the clustering of patient samples on the basis of a combination of sequence data from several TCR and/or B‐cell receptor (BCR) loci, an approach beyond the scope of the present study.

While the exact features mediating sample clustering are difficult to define with a machine learning method such as ours, the advantage of our method is that it provides a holistic analysis of DNA‐based TCRR sequences that considers similar, but non‐identical, CDR3 sequences, as being more closely related to each other than any two CDR3 sequences chosen at random. This contrasts with methodologies that simply determine clonal status or search for specific clones or motifs within the TCRR, which do not take account of the presence of closely related CDR3 sequences [4, 23, 25, 26, 27, 28, 29, 30, 31, 32, 33]. Furthermore, methodologies that search for one or a small number of specific TCR clones risk generating false‐negative results, if a critical, but low frequency, sequence is missed, leading to poor sensitivity.

While *k*mers have been used to analyse analogous BCR sequences, in two previous studies [34, 35] neither study employed *k*mers for holistic CDR3 analysis or as the basis of sample classification. The accurate clustering achieved by our method suggests that there is a greater degree of similarity in the *k*mer usage, and thus in the CDR3 sequences from which these *k*mers derive, between patients with CeD than between non‐CeD controls. This observation is in keeping with an immune response to a stereotyped set of antigens in the CeD patients. Further work is required to explore the exact *k*mer patterns underpinning this similarity and identification of these sequences has the potential to provide insight into the underlying immunological mechanisms of CeD. For example, holistic *k*mer‐based TCR analysis could be used as a method to identify consensus sequences within the TCRR of a cohort of CeD patients, with the numbers of separate groups of immunoreceptor consensus sequences giving an indication of the likely numbers of different epitopes being recognised in the condition.

Biological understanding and computational methods for γδ T‐cells are not yet well enough developed to predict likely epitopes/antigens bound from the TCR CDR3 sequences alone. Indeed, relatively little is known about γδ T‐cells' antigen binding mechanisms, unlikely αβ T‐cells, which are known to recognise short peptide antigens bound to MHC molecules, with the TCR generally contacting both the peptide and the MHC protein. The ability of short *k*mer sequences, derived from the CDR3 sequences of γδ T‐cells, to separate CeD from normal biopsies indicates that CDR3 sequences are likely to be very important in γδ T‐cell‐antigen binding or other γδ T‐cell interactions in CeD. This observation fits well with a recent study of CeD duodenum, demonstrating depletion of resident duodenal Vγ4^+^/Vδ1^+^ intraepithelial lymphocytes (IELs), with semi‐invariant TCRs, and their replacement with gluten‐sensitive, interferon‐γ‐producing Vδ1^+^ IELs bearing T‐cell receptors (TCRs) with CDR3 motifs that are shared both within and between CeD patients' TRD repertoires. Canonical sets of *k*mers from these shared CDR3 motifs are likely to be a key feature in the CeD patient TCRR, detected by our classification algorithm [4].

It likely that a major reason for the success of our algorithm is the fact that it compares non‐CeD and CeD duodenum, rather than simply looking for predefined features of CeD duodenum. It is thus able to detect a signal based both on the loss of the semi‐invariant Vγ4^+^/Vδ1^+^ TCRs of the innate‐like γδ T‐cell population and on the development of the antigen‐driven Vδ1^+^ γδ T‐cell population. The loss of these semi‐invariant Vγ4^+^/Vδ1^+^ TCRs may contribute to the fact that we see an unexpected increase in TCR diversity in CeD, rather than the decrease one might expect if there is an evolving clonal response to gluten. The relatively poorly characterised, innate‐like Vγ4^+^/Vδ1^+^ IELs are thought to maintain homeostasis in the local small intestinal microenvironment, either by eliminating virus‐infected or malignant cells in response to innate signals, or by promoting tissue healing via the production of growth factors [4]. Their presence appears to be key in maintaining a gluten‐tolerant IEL population. Therefore, detecting loss of Vγ4^+^/Vδ1^+^ IELs may be as important as detecting the novel gluten‐sensitive Vδ1^+^ IELs in our ability to classify samples as CeD or non‐CeD on the basis of TCRR.

Commensurate with our observation that duodenal biopsies from CeD patients on a GFD are correctly classified on the basis of TRG TCRR, exclusion of dietary gluten in the study by Mayassi *et al* was insufficient to reconstitute the physiological Vγ4^+^/Vδ1^+^ IEL population [4]. Our ability to classify duodenal biopsies from CeD patients on a GFD correctly is also in keeping with reports of persistent elevation of intraepithelial γδ T‐cells [2, 3, 4, 5, 6] in CeD patients, even on a GFD. These data show that CeD‐associated γδ T‐cells do not recirculate away from the small intestine in the absence of dietary gluten. The observed persistence of CeD‐associated γδ T‐cells in the duodenum, without gluten ingestion, indicates that TRG and TRD may be the most appropriate TCR loci to analyse to determine gluten sensitivity (CeD) status, tests which are otherwise confounded by insufficient gluten intake. This biological phenomenon appears to underpin the success of our potential novel diagnostic approach to the condition.

In summary, we have developed a machine learning algorithm that following further testing on a larger cohort, could be used for CeD diagnosis, regardless of dietary gluten status, which uses TCR sequencing methodology amenable to current histopathological and molecular diagnostic workflows. Our novel, machine learning‐based bioinformatic approach is generalisable to sequences from all four TCR and all three BCR loci, which are obtained using any sequencing platform. Thus, this approach might similarly be applied to the prediction of diagnosis or prognosis in other conditions mediated by the adaptive immune response. These might include other autoimmune or immune‐mediated inflammatory conditions, allergic reactions, and possibly immune responses to both infections, such as SARS‐CoV‐2, and cancers.

## Author contributions statement

EJS and AF designed and conceived the study. KD and SCE selected samples. MSS, SCE and NC performed experiments. OE, RP, MSS, ADF and AF analysed the data. ADF, AF, MEBF, PK and EJS wrote the manuscript. All the authors reviewed and approved the final manuscript.

## Data availability statement

The classification algorithm, implemented in R scripts, is available from Zenodo (http://doi.org/10.5281/zenodo.3964131).

## Supporting information


**Supplementary materials** **and** **methods**

**Figure S1.** Potentially confounding variables in TRD cannot reliably separate coeliac disease samples from control samples
**Figure S2.** TRD CDR3 length analysis cannot separate coeliac disease samples from control samples
**Figure S3.** Leave‐one‐out cross‐validation for TRD using non‐positional 4mers
**Figure S4.** Potentially confounding variables in TRG cannot reliably separate coeliac disease samples from control samples
**Figure S5.** TRG CDR3 length analysis cannot separate coeliac disease samples from control samples
**Figure S6.** Leave‐one‐out cross‐validation for TRG using positional 5mers
**Table S1.** Details of all study subjects and criteria for inclusion
**Table S2.** Properties of raw and processed TRD and TRG sequence data
**Table S3.** Training accuracy, sensitivity, and specificity results of non‐positional 4mer cluster analysis for TRD
**Table S4.** Training accuracy, sensitivity, and specificity results of positional 7mer cluster analysis for TRD
**Table S5.** Training accuracy, sensitivity, and specificity results of CDR3 cluster analysis for TRD
**Table S6.** Training accuracy, sensitivity, and specificity results of non‐positional 4mer cluster analysis for TRD DNA after random downsampling to the minimum read count
**Table S7.** Training accuracy, sensitivity, and specificity results of non‐positional 4mer cluster analysis for TRD DNA after collapsing of CDR3 sequence data of all the samples in the cohort to a frequency of 1 for every CDR3 sequence
**Table S8.** Training accuracy, sensitivity, and specificity results of non‐positional 4mer cluster analysis for TRD DNA after random downsampling to the minimum read count and collapsing of CDR3 sequence data of all the samples in the cohort to a frequency of 1 for every CDR3 sequence
**Table S9.** Training accuracy, sensitivity, and specificity results of positional 5mer cluster analysis for TRG
**Table S10.** Training accuracy, sensitivity and specificity results of non‐positional 4mer cluster analysis for TRG
**Table S11.** Training accuracy, sensitivity, and specificity results of CDR3 cluster analysis for TRG
**Table S12.** Training accuracy, sensitivity, and specificity results of positional 5mer cluster analysis for TRG, with samples divided into two groups by age
**Table S13.** Training accuracy, sensitivity, and specificity results of positional 5mer cluster analysis for TRG DNA after random downsampling to the minimum read count
**Table S14.** Training accuracy, sensitivity, and specificity results of positional 5mer cluster analysis for TRG DNA after collapsing of CDR3 sequence data of all the samples in the cohort to a frequency of 1 for every CDR3 sequence
**Table S15.** Training accuracy, sensitivity, and specificity results of positional 5mer cluster analysis for TRG DNA after random downsampling to the minimum read count and collapsing of CDR3 sequence data of all the samples in the cohort to a frequency of 1 for every CDR3 sequence
