# Supplementary material for: Classification of intestinal T‐cell receptor repertoires using machine learning methods can identify patients with coeliac disease regardless of dietary gluten status
Source: J Pathol. 2021 Jan 6;253(3):279–91. doi: 10.1002/path.5592 (PMC7898595; doi:10.1002/path.5592)
Supplement: Supplementary file 1 — Supplementary materials and methods [file PATH-253-279-s001.docx]

**Classification of intestinal T-cell receptor repertoires using machine learning methods can identify patients with coeliac disease regardless of dietary gluten status**

AD Foers, MS Shoukat, *et al. J Pathol* DOI: 10.1002/path.5592

**Supplementary materials and methods**

Reference numbers refer to the main text list

**Identification of optimal classification from a clustering hierarchy**

We assume that a clustering hierarchy partitions the data into two clusters, corresponding to healthy patients and those with coeliac disease. The hierarchies consistently show that the coeliac patients tend to cluster first, since they are more similar to each other, whereas samples from normal patients are less closely related and are added to a single large cluster later. Therefore, we partition the tree into two clusters by choosing a merge (node) and defining the cluster created at that merge as a single cluster (or, alternatively, all leaves descending from that node as a single cluster), then assigning all other samples (leaves) into a single cluster. The two clusters are then labelled either coeliac or normal according to the true classification of the majority of samples in each cluster. If both clusters contain equal numbers of CeD and non-CeD samples, then clusters are labelled randomly. Sensitivity is then calculated as the fraction of coeliac samples correctly labelled, specificity as the fraction of normal samples correctly labelled, and accuracy as the fraction of all samples correctly labelled. The cluster configuration from the hierarchy which has the highest accuracy is then chosen as the single configuration to represent that hierarchy.

We note that this approach to defining two clusters relies upon the structure we see in our hierarchies, which typically consists of a single large cluster formed by first merging coeliac samples, then normal samples. This particular structure avoids the problems of determining the optimal number of clusters and of preference being given to small but sensitive clusters. Visual inspection of any hierarchies with particularly high or low accuracies was used in order to ensure that the structure of these hierarchies was consistent with this assumption.

**Parameter optimisation**

The algorithm parameters (Figure 1) to be optimised are input type (positional *k*mers, non-positional *k*mers, full-length CDR3), value of *k* (length of *k*mer), and principal components used. The clustering hierarchies from all possible combinations of algorithm parameters are generated (supplementary material, Tables S3–S15), and the optimal parameter set is determined as follows.

The optimal *k*mer length (except when using full-length CDR3s) is determined first and is defined as the *k*mer length that has the highest accuracy when combined with a single set of PCs. However, there are often ties with multiple *k*mer lengths achieving the same accuracy when using at least one set of PCs. In this case, the *k*mer length with the largest number of PC combinations that give the highest accuracy is defined as optimal. For instance, if using 4mers resulted in 100 PC combinations achieving 100% accuracy, whereas using 5mers resulted in 95 combinations achieving 100% accuracy, then 4mers would be chosen as the optimal *k*mer length.

We then define the optimal PC combination (conditional on the optimal *k*mer length) as the combination that gives the highest accuracy and in the case of ties, the greatest separation (known as a mutual reachability distance) between the diagnostic groups. Similarly, we define the optimal input type (conditional on the optimal *k*mer length and PC combination) as the one that gives the highest accuracy and in the case of ties, the greatest separation.

When defining accuracy, the term ‘training accuracy’ is used when the entire sample cohort is used in the initial semi-supervised learning process (supplementary material, Tables S3–S15), while ‘testing accuracy’ refers to values obtained by means of leave-one-out cross-validation (supplementary material, Figures S3 and S6).

*P* values assessing training accuracy (supplementary material, Tables S3–S15) were calculated using Fisher’s exact test and adjusted for multiple comparisons, using the Benjamini–Hochberg false discovery rate (BH) and Bonferroni methods [45,46].

**Shannon diversity**

Shannon diversity (Shannon entropy) indices [18,47] were calculated using the vegan R package [48]. To account for different sequencing depths between samples, Shannon indices were calculated from a random subsample of reads equal to the lowest total number of numbers of aligned CDR3 reads within an individual dataset. This was repeated 1000 times and average Shannon indices were calculated. The statistical significance of differences in Shannon diversity between groups was determined using a permutation test [49], with 1000 permutations (supplementary material, Figures S1A and S4A). This makes no assumptions about any underlying distributions and generates an empirical distribution by permuting sample labels, then recalculating the statistic of interest.

**CDR3 length analyses**

Histograms of frequencies of CDR3 sequences of different lengths were constructed (supplementary material, Figures S2A and S5A). Quadratic regression [50] was used to determine how close the fit of the CDR3 lengths was to a theoretical Gaussian distribution for each sample, with more Gaussian distributions having a coefficient of determination, *R*^2^, closer to 1, while less Gaussian distributions have an *R*^2^ value closer to 0. *R*^2^ values for the two groups of samples were compared (supplementary material, Figures S2B and S5B) using a two-tailed Mann–Whitney *U*-test. The mean CDR3 length for each sample was plotted (supplementary material, Figures S1B and S2B), and comparisons were made between CeD and non-CeD samples with a two-tailed Student’s *t*-test. Finally, cumulative CDR3 length distributions were plotted for the two sample groups (supplementary material, Figures S1C and S2C) and compared by means of a Kolmogorov–Smirnov test [51], generating a Kolmogorov–Smirnov *D* statistic (KSD) and a *P* value. We specifically elected to use a non-parametric test that was able to compare frequencies across the full range of CDR3 lengths.

**Assignment to two groups on the basis of age**

Age can influence TCR profiles [18], and as there was a significant difference in the age of the CeD and non-CeD cohorts (Table 1 and supplementary material, Table S1), we investigated the effects of age on sample classification. The 33 CeD patients were stratified by age and split into a group comprising the 17 older patients and a group comprising the 16 younger patients. Here, a positional *k*mer length of 5 performed best, with just one principal component combination able to classify high from low age with 79% accuracy (supplementary material, Figure S4G). However, this was not statistically significant (supplementary material, Table S12), indicating that patient age is not driving sample classification.

**Downsampling to give equal read counts**

When our *k*mer-based clustering algorithm was run, the *k*mer count for each sample was normalised, so that absolute CDR3 frequencies did not confound the analysis. To determine the effect of read count on sample classification, reads from each patient were downsampled to the level of the sample with the lowest number of reads to give equal read counts, set at the level of the minimum read count obtained for any sample in the cohort (TRD = 7256; TRG = 16 250). Following downsampling, CeD and non-CeD samples were classified with comparable accuracy to that in the primary analysis (supplementary material, Figures S1G and S4H and Tables S6 and S13).

**Collapsing CDR3 frequencies to 1**

In any given patient sample, a number of CDR3 amino acid sequences were present multiple times, likely biological reasons for which include (1) prior clonal expansion of T-cells as a response to antigen (potentially including gluten and self-antigen) and (2) identical TCR clonotypes at amino acid level being derived from differing nucleic acid sequences because of triplet code redundancy, such amino-acid-identical TCR clonotypes likely being present because of the relevance of their specific TCR to antigen binding. However, CDR3 amino acid sequences being present multiple times cannot be distinguished from the effect of PCR duplicates. The PCR method used has been extensively validated and has limited amplification bias [22], and therefore the ratio of PCR duplicates should be closely proportional to the ratio of the TCR sequences in the DNA template. This minimal amplification bias, combined with normalisation of read count for each sample, prior to generation of *k*mers, means that PCR duplicates are unlikely to confound any part of the analysis. Notwithstanding, to test the robustness of our method, largely by decreasing the contributions of the most frequent and thus likely the most relevant TCR sequences, but also by removing any possible PCR duplicates, we removed the CDR3 frequency metric, collapsing all frequencies to 1 and repeated the cluster analysis (supplementary material, Figures S1H and S4I and Tables S7 and S14). There was little decrease in training accuracy, confirming the robustness of our method. To maximise stringency, collapsing of CDR3 frequencies to 1 was also combined with downsampling of reads, again being associated with little decrease in training accuracy (supplementary material, Figures S1I and S4J and Tables S8 and S15).

**Statistical analysis of patient demographic and laboratory test data**

*P* values were calculated using a two-tailed Student’s *t*-test or Fisher’s exact test as indicated.
