# Supplementary material for: Classification of intestinal T‐cell receptor repertoires using machine learning methods can identify patients with coeliac disease regardless of dietary gluten status
Source: J Pathol. 2021 Jan 6;253(3):279–91. doi: 10.1002/path.5592 (PMC7898595; doi:10.1002/path.5592)
Supplement: Supplementary file 2 — Figure S1. Potentially confounding variables in TRD cannot reliably separate coeliac disease samples from control samples Figure S2. TRD CDR3 length analysis cannot separate coeliac disease samples from control samples Figure S3. Leave‐one‐out cross‐validation for TRD using non‐positional 4mers Figure S4. Potentially confounding variables in TRG cannot reliably separate coeliac disease samples from control samples Figure S5. TRG CDR3 length analysis cannot separate coeliac disease samples from control samples Figure S6. Leave‐one‐out cross‐validation for TRG using positional 5mers Table S1. Details of all study subjects and criteria for inclusion Table S2. Properties of raw and processed TRD and TRG sequence data Table S3. Training accuracy, sensitivity, and specificity results of non‐positional 4mer cluster analysis for TRD Table S4. Training accuracy, sensitivity, and specificity results of positional 7mer cluster analysis for TRD Table S5. Training accuracy, sensitivity, and specificity results of CDR3 cluster analysis for TRD Table S6. Training accuracy, sensitivity, and specificity results of non‐positional 4mer cluster analysis for TRD DNA after random downsampling to the minimum read count Table S7. Training accuracy, sensitivity, and specificity results of non‐positional 4mer cluster analysis for TRD DNA after collapsing of CDR3 sequence data of all the samples in the cohort to a frequency of 1 for every CDR3 sequence Table S8. Training accuracy, sensitivity, and specificity results of non‐positional 4mer cluster analysis for TRD DNA after random downsampling to the minimum read count and collapsing of CDR3 sequence data of all the samples in the cohort to a frequency of 1 for every CDR3 sequence Table S9. Training accuracy, sensitivity, and specificity results of positional 5mer cluster analysis for TRG Table S10. Training accuracy, sensitivity and specificity results of non‐positional 4mer cluster analysis for TRG Table S11. Training accuracy, sensitivity, [file PATH-253-279-s002.zip › path5592-sup-SuppFigTableLegs.docx]

**Classification of intestinal T-cell receptor repertoires using machine learning methods can identify patients with coeliac disease regardless of dietary gluten status**

AD Foers, MS Shoukat, *et al. J Pathol* DOI: 10.1002/path.5592

**Supplementary Figure and Table legends**

**Figure S1. Potentially confounding variables in TRD cannot reliably separate coeliac disease samples from control samples**. (A) Substantially greater TRD clonal diversity (higher Shannon diversity) is observed in CeD than in non-CeD biopsies [*P* value (based on a permutation test (10 000 permutations) = 0.0 (highly significant))]. This has two implications: Firstly, it confirms that the clustering of CeD samples is not based on a clonal tendency, as CeD samples show greater TRD repertoire diversity. Secondly, it is likely that this difference in diversity, which is presumably due to the increased numbers of T-cells per unit volume of biopsy and thus per unit mass of DNA in CeD biopsies, may be captured by one or more PCs used to mediate clustering of samples. Thus, because of our novel methodology, our AI algorithm has the potential to detect additional parameters of the data beyond simple *k*mer sequence frequencies, as it is likely also to take into account clonal diversity when separating samples by diagnosis. (B) Mean TRD CDR3 lengths do not differ significantly between CeD and non-CeD biopsies. (C) Cumulative TRD CDR3 lengths do not differ significantly between CeD and non-CeD biopsies, as assessed using the Kolmogorov–Smirnov’s *D* statistic (KSD) and *P* value. (D) Hierarchical clustering on the basis of combinations of V segment usage in the sequence data could not classify patient samples by diagnosis. (E) Hierarchical clustering on the basis of combinations of J segment usage in the sequence data could not classify patient samples by diagnosis. (F) Accuracy of best performing cluster analyses, for TRD non-positional 4mers, for 100 randomly labelled permutations of the patient data, giving a mean best score of 17.1/22 (77.8%) training accuracy across all permutations, compared with 22/22 (100%) for the training dataset (*p*< 0.01 using a permutation test). (G) Training accuracy results of non-positional 4mer cluster analysis for TRD after random downsampling to the minimum read count (7256) of all the samples in the cohort, in order to demonstrate that read count does not act as a confounding variable in sample classification with our algorithm. (H) Training accuracy results of non-positional 4mer cluster analysis for TRD after collapsing the frequency of every unique CDR3 sequence to 1 for all the samples in the cohort, in order to demonstrate that PCR duplicates are not acting as a confounding variable in sample classification with our algorithm. This is likely to be an excessively stringent approach, in that counts above 1 for a particular CDR3 sequence may be evidence of an immune response. (I) Training accuracy results of non-positional 4mer cluster analysis for TRD after random downsampling to the minimum read count (7256) of all the samples in the cohort and collapsing the frequency of every unique CDR3 sequence to 1 for all the samples in the cohort, in order to demonstrate that neither PCR duplicates nor read count is acting as a confounding variable in sample classification with our algorithm. Sequence data that support the findings of this study have been deposited in the NCBI SRA database with the accession code SUB7836297.

**Figure S2. TRD CDR3 length analysis cannot separate coeliac disease samples from control samples.** (A) To assess TRD CDR3 length distributions, CeD length versus frequency distributions are shown across the patient cohort. The normality of each distribution is estimated by fitting a Gaussian curve and calculating the *R*^2^ value. (B) Differences in *R*^2^ values between CeD and non-CeD samples were assessed by a Mann–Whitney two-tailed test. Error bars = SEM.

**Figure S3. Leave-one-out cross-validation for TRD using non-positional 4mers.** The sample removed and re-added is shown in green in each panel. Testing accuracy was calculated as the percentage of times the sample shown in green clustered correctly.

**Figure S4. Potentially confounding variables in TRG cannot reliably separate coeliac disease samples from control samples.** (A) As for TRD (supplementary material, Figure S1A), substantially greater TRG clonal diversity (higher Shannon diversity) is observed in CeD than in normal biopsies [*P* value (based on a permutation test (10 000 permutations) =0.0 (highly significant))], with similar implications. (B) Mean TRG CDR3 lengths do not differ significantly between CeD and non-CeD biopsies. (C) Cumulative TRG CDR3 lengths do not differ significantly between CeD and non-CeD biopsies, as assessed using Kolmogorov–Smirnov’s *D* statistic (KSD; methodology as for supplementary material, Figure S1). (D) Hierarchical clustering on the basis of combinations of V segment usage in the sequence data could not classify patient samples by diagnosis. (E) Hierarchical clustering on the basis of combinations of J segment usage in the sequence data could not classify patient samples by diagnosis. (F) Accuracy of best performing cluster analyses, for TRG positional 5mers, for 100 randomly labelled permutations of the patient data, giving a mean best score of 39.3/54 (72.7%) training accuracy across all permutations, compared with 51/54 (94.4%) for the training dataset (*p*< 0.01 using a permutation test). (G) Training accuracy results of positional 5mer cluster analysis for TRG are poor, when samples are divided into two groups based on age (younger 16 versus older 17), indicating that separation by diagnosis is specific (see also supplementary material, Table S12). (H) Training accuracy results of non-positional 4mer cluster analysis for TRD after random downsampling to the minimum read count (7256) of all the samples in the cohort, in order to demonstrate that read count does not act as a confounding variable in sample classification with our algorithm. (I) Training accuracy results of non-positional 4mer cluster analysis for TRD after collapsing the frequency of every unique CDR3 sequence to 1 for all the samples in the cohort, in order to demonstrate that PCR duplicates are not acting as a confounding variable in sample classification with our algorithm. This is likely to be an excessively stringent approach, in that counts above 1 for a particular CDR3 sequence may be evidence of an immune response. (J) Training accuracy results of non-positional 4mer cluster analysis for TRD after random downsampling to the minimum read count (7256) of all the samples in the cohort and collapsing the frequency of every unique CDR3 sequence to 1 for all the samples in the cohort, in order to demonstrate that neither PCR duplicates nor read count is acting as a confounding variable in sample classification with our algorithm.

**Figure S5. TRG CDR3 length analysis cannot separate coeliac disease samples from control samples.** To assess TRG CDR3 length distributions, CeD length versus frequency distributions were compared across (A) the initial and (B) additional longitudinal GFD patient cohorts. The normality of each distribution is estimated by fitting a Gaussian curve and calculating the *R*^2^ value. (C) Differences in *R*^2^ values between the initial CeD and non-CeD samples were assessed by a Mann–Whitney two-tailed test. Error bars = SEM.

**Figure S6.** Leave-one-out cross-validation for TRG using positional 5mers. The sample removed and re-added is shown in green in each panel. Testing accuracy was calculated as the percentage of times the sample shown in green clustered correctly.

**Table S1.** Details of all study subjects and criteria for inclusion

**Table S2.** Properties of raw and processed TRD and TRG sequence data

**Table S3.** Training accuracy, sensitivity, and specificity results of non-positional 4mer cluster analysis for TRD. *P* values were calculated using Fisher’s exact test and are adjusted for multiple comparisons using the Benjamini–Hochberg false discovery rate and Bonferroni procedures

**Table S4.** Training accuracy, sensitivity, and specificity results of positional 7mer cluster analysis for TRD. *P* values were calculated as in supplementary material, Table S3

**Table S5.** Training accuracy, sensitivity, and specificity results of CDR3 cluster analysis for TRD

**Table S6.** Training accuracy, sensitivity, and specificity results of non-positional 4mer cluster analysis for TRD after random downsampling to the minimum read count (7256) of all the samples in the cohort, in order to demonstrate that read count does not act as a confounding variable in sample classification with our algorithm, by comparison with supplementary material Table S3. *P* values were calculated as in supplementary material, Table S3

**Table S7.** Training accuracy, sensitivity, and specificity results of non-positional 4mer cluster analysis for TRD after collapsing of CDR3 sequence data of all the samples in the cohort to a frequency of 1 for every CDR3 sequence, in order to eliminate the possibility of PCR duplicates and/or amplification bias acting as a confounding variable in sample classification with our algorithm, by comparison with supplementary material, Table S3. *P* values were calculated as in supplementary material, Table S3

**Table S8.** Training accuracy, sensitivity, and specificity results of non-positional 4mer cluster analysis for TRD after random downsampling to the minimum read count (7256) and collapsing of CDR3 sequence data of all the samples in the cohort to a frequency of 1 for every CDR3 sequence, in order to eliminate the effect of read count and the possibility of PCR duplicates and/or amplification bias acting as a confounding variable in sample classification with our algorithm, by comparison with supplementary material, Table S3. *P* values were calculated as in supplementary material, Table S3

**Table S9.** Training accuracy, sensitivity, and specificity results of positional 5mer cluster analysis for TRG. *P* values were calculated as in supplementary material, Table S3

**Table S10.** Training accuracy, sensitivity, and specificity results of non-positional 4mer cluster analysis for TRG. *P* values were calculated as in supplementary material, Table S3

**Table S11.** Training accuracy, sensitivity, and specificity results of CDR3 cluster analysis for TRD. *P* values were calculated as in supplementary material, Table S3

**Table S12.** Training accuracy, sensitivity, and specificity results of positional 5mer cluster analysis for TRG coeliac patient samples only, with samples divided into the older (*n* = 17) and younger (*n* = 16) patient groups, rather than two groups classified by diagnosis, for comparison with supplementary material, Table S10. *P* values were calculated as in supplementary material, Table S3

**Table S13.** Training accuracy, sensitivity, and specificity results of positional 5mer cluster analysis for TRG after random downsampling to the minimum read count (16 250) of all the samples in the cohort, in order to demonstrate that read count does not act as a confounding variable in sample classification with our algorithm, by comparison with supplementary material, Table S10. *P* values were calculated as in supplementary material, Table S3

**Table S14.** Training accuracy, sensitivity, and specificity results of positional 5mer cluster analysis for TRG after collapsing of CDR3 sequence data of all the samples in the cohort to a frequency of 1 for every CDR3 sequence, in order to eliminate the possibility of PCR duplicates and/or amplification bias acting as a confounding variable in sample classification with our algorithm, by comparison with supplementary material, Table S10. *P* values were calculated as in supplementary material, Table S3

**Table S15.** Training accuracy, sensitivity, and specificity results of positional 5mer cluster analysis for TRG after random downsampling to the minimum read count (16 250) and collapsing of CDR3 sequence data of all the samples in the cohort to a frequency of 1 for every CDR3 sequence, in order to eliminate the effect of read count and the possibility of PCR duplicates and/or amplification bias acting as a confounding variable in sample classification with our algorithm, by comparison with supplementary material, Table S10. *P* values were calculated as in supplementary material, Table S3
