# Supplementary figures and images for: Classification of intestinal T‐cell receptor repertoires using machine learning methods can identify patients with coeliac disease regardless of dietary gluten status
Source: J Pathol. 2021 Jan 6;253(3):279–91. doi: 10.1002/path.5592 (PMC7898595; doi:10.1002/path.5592)

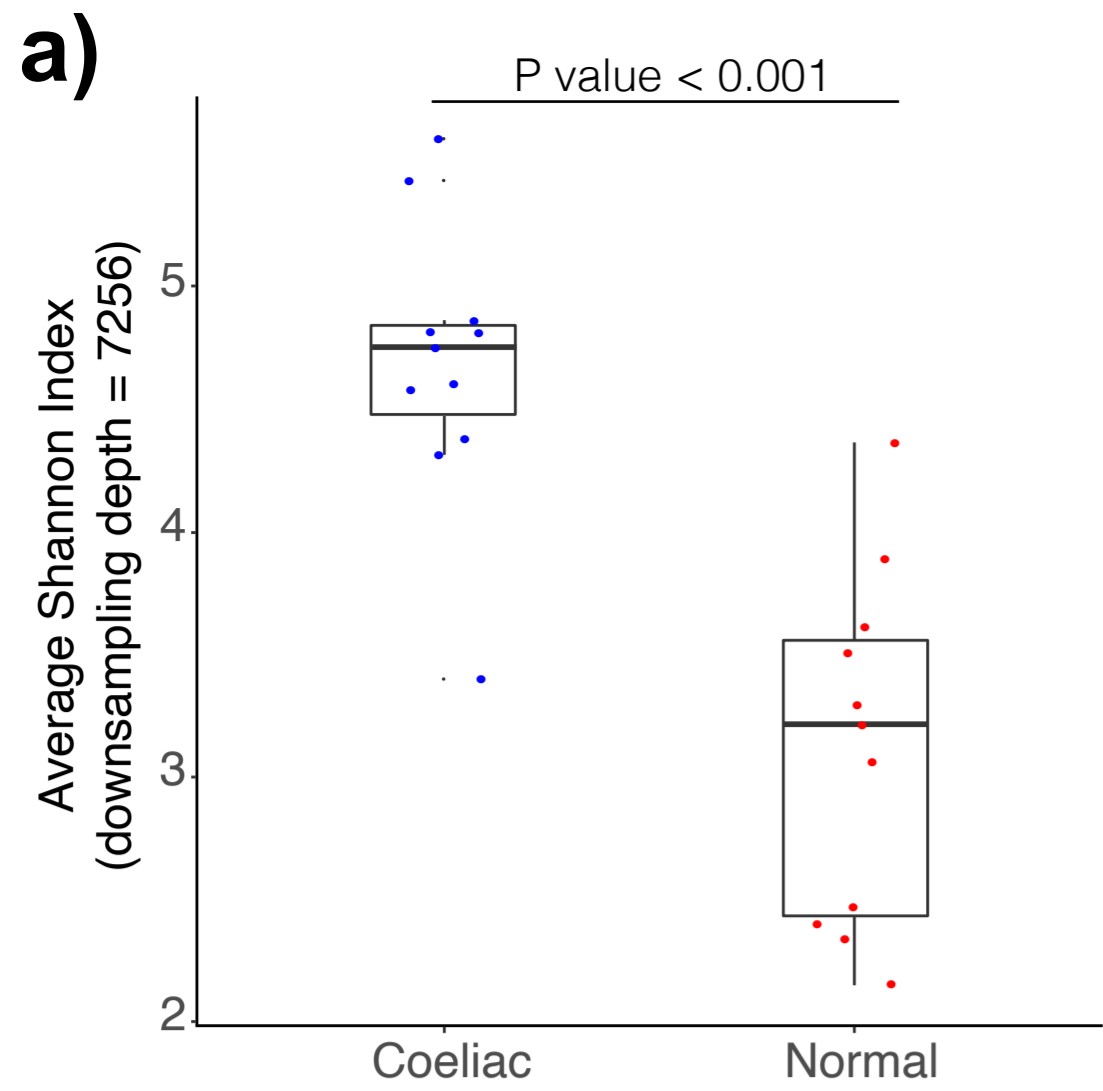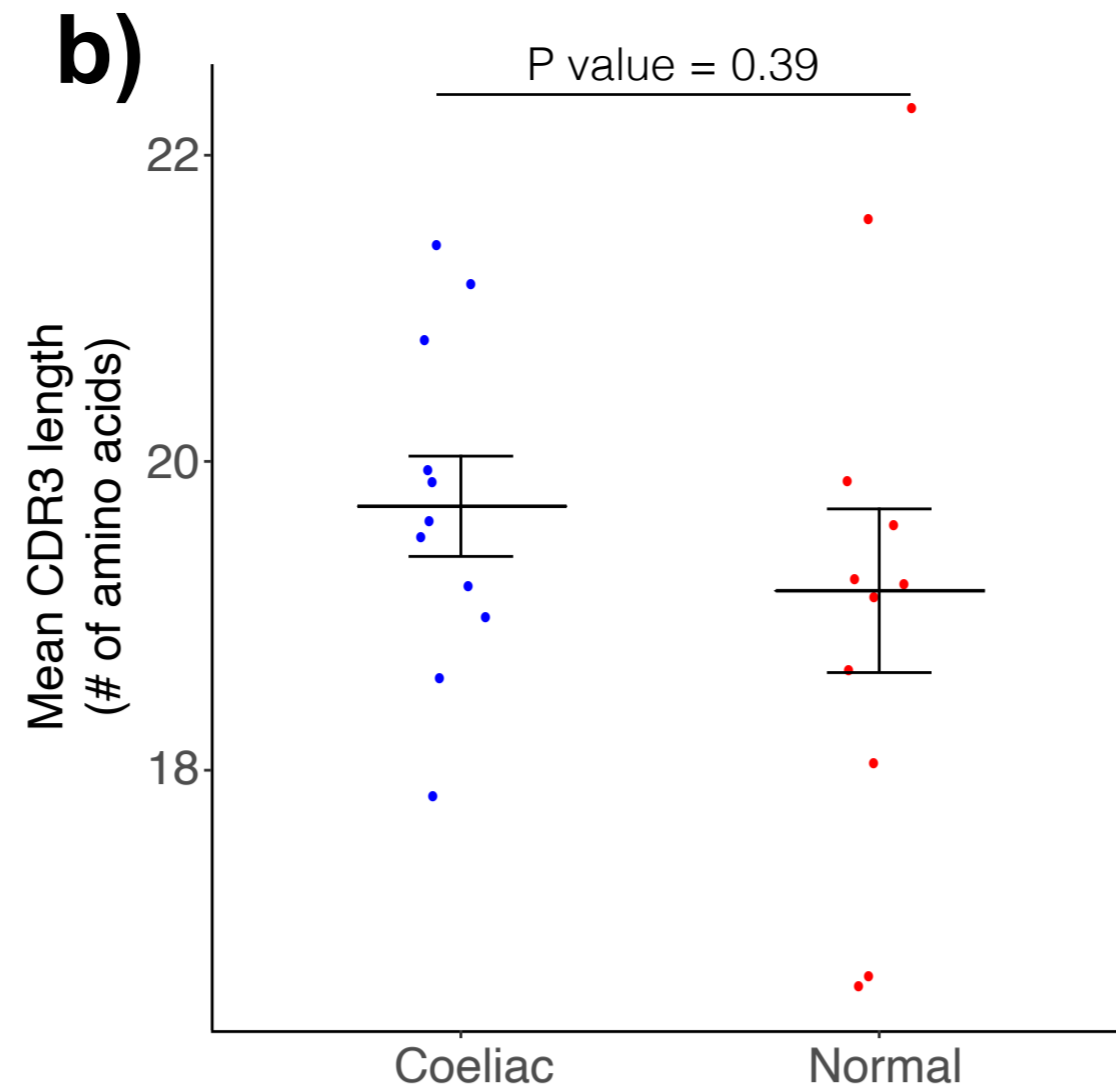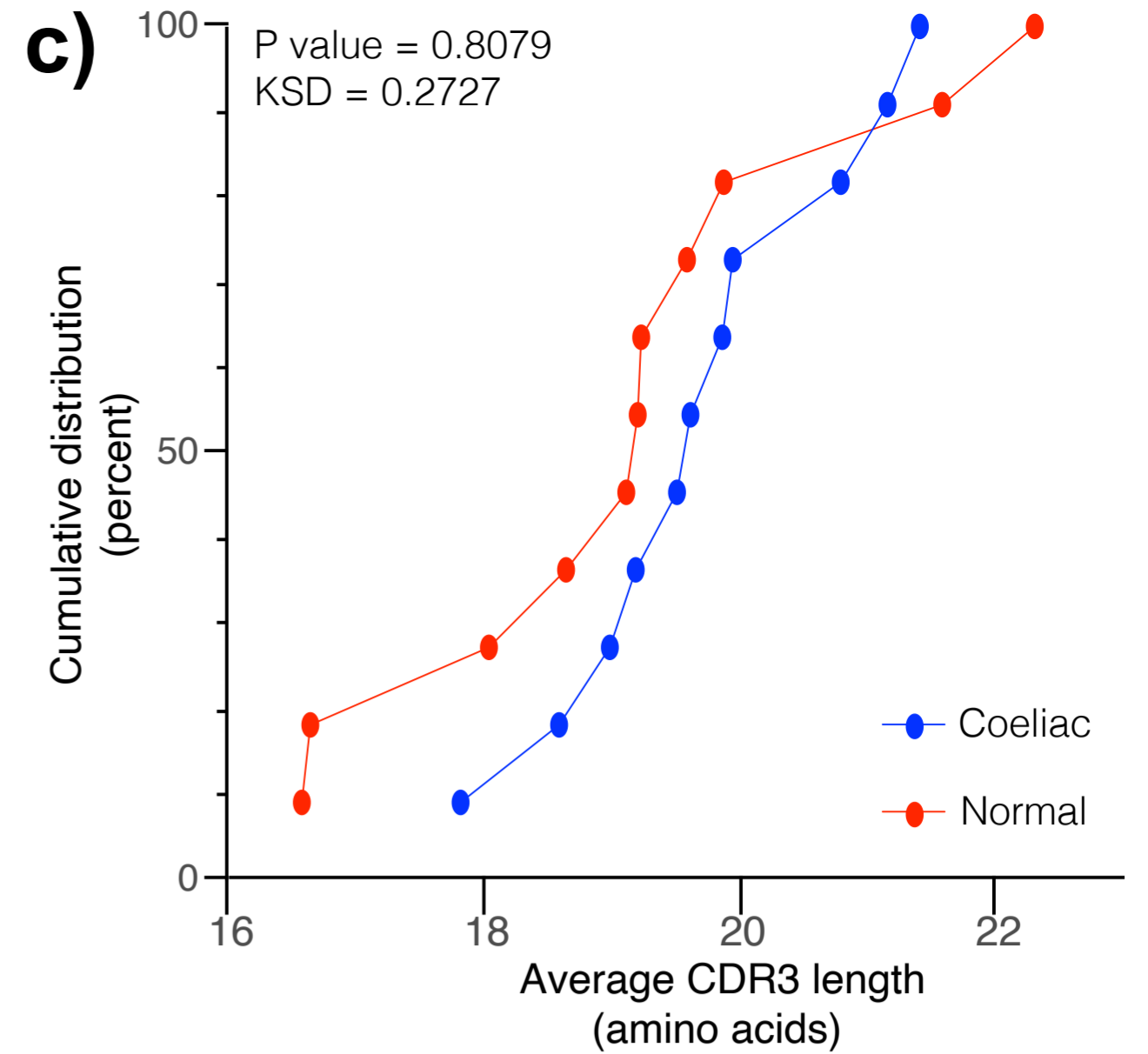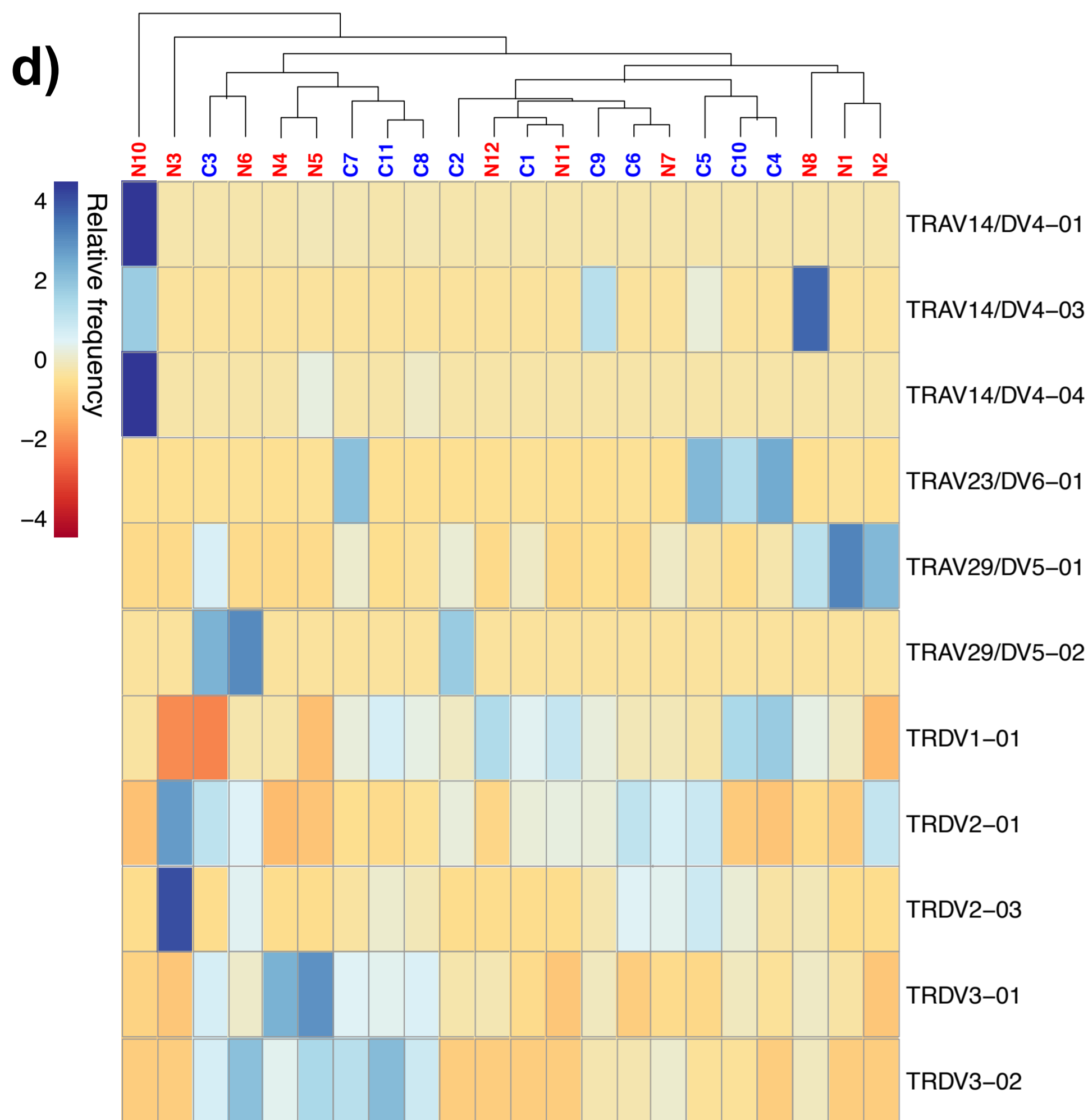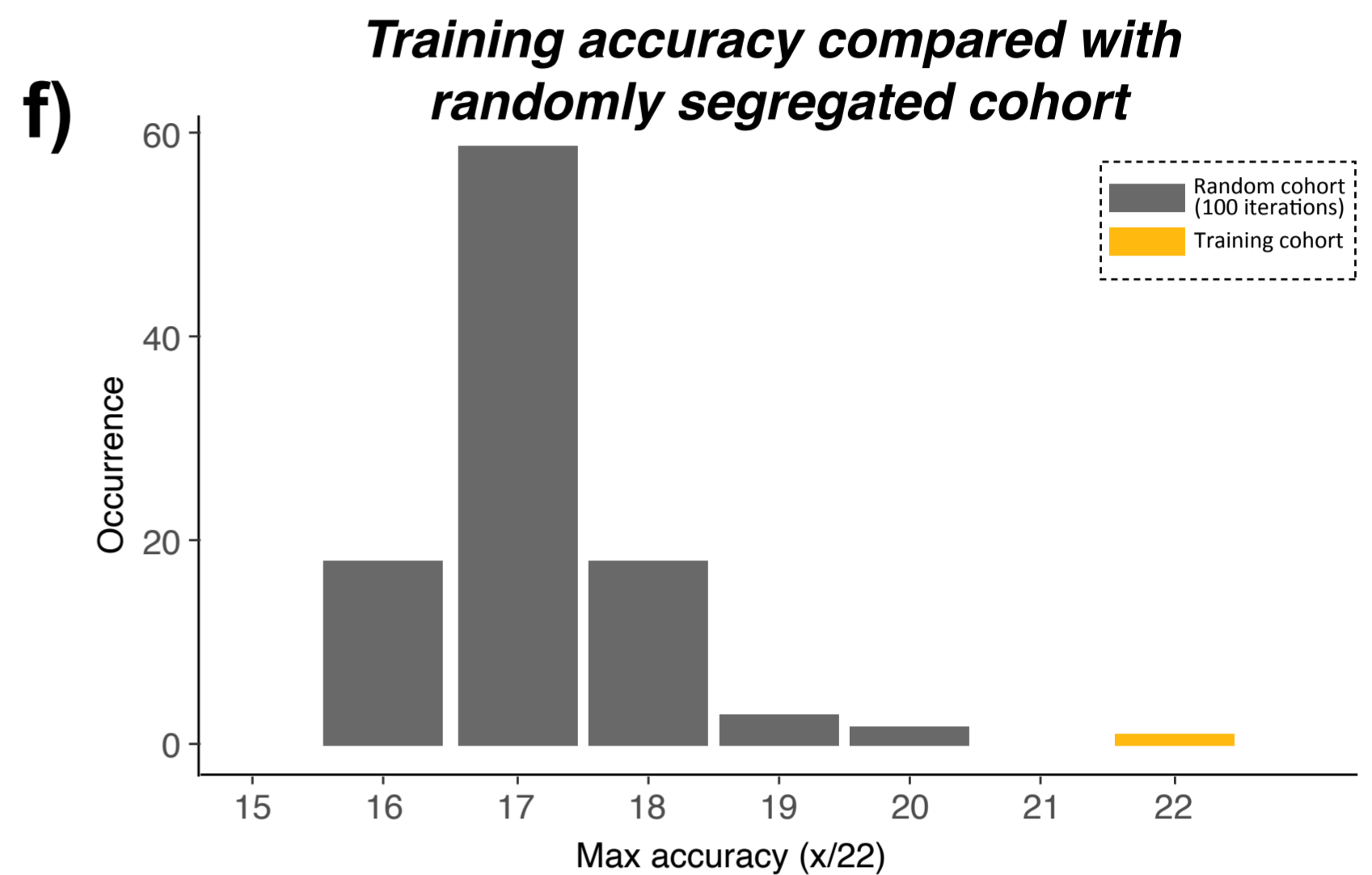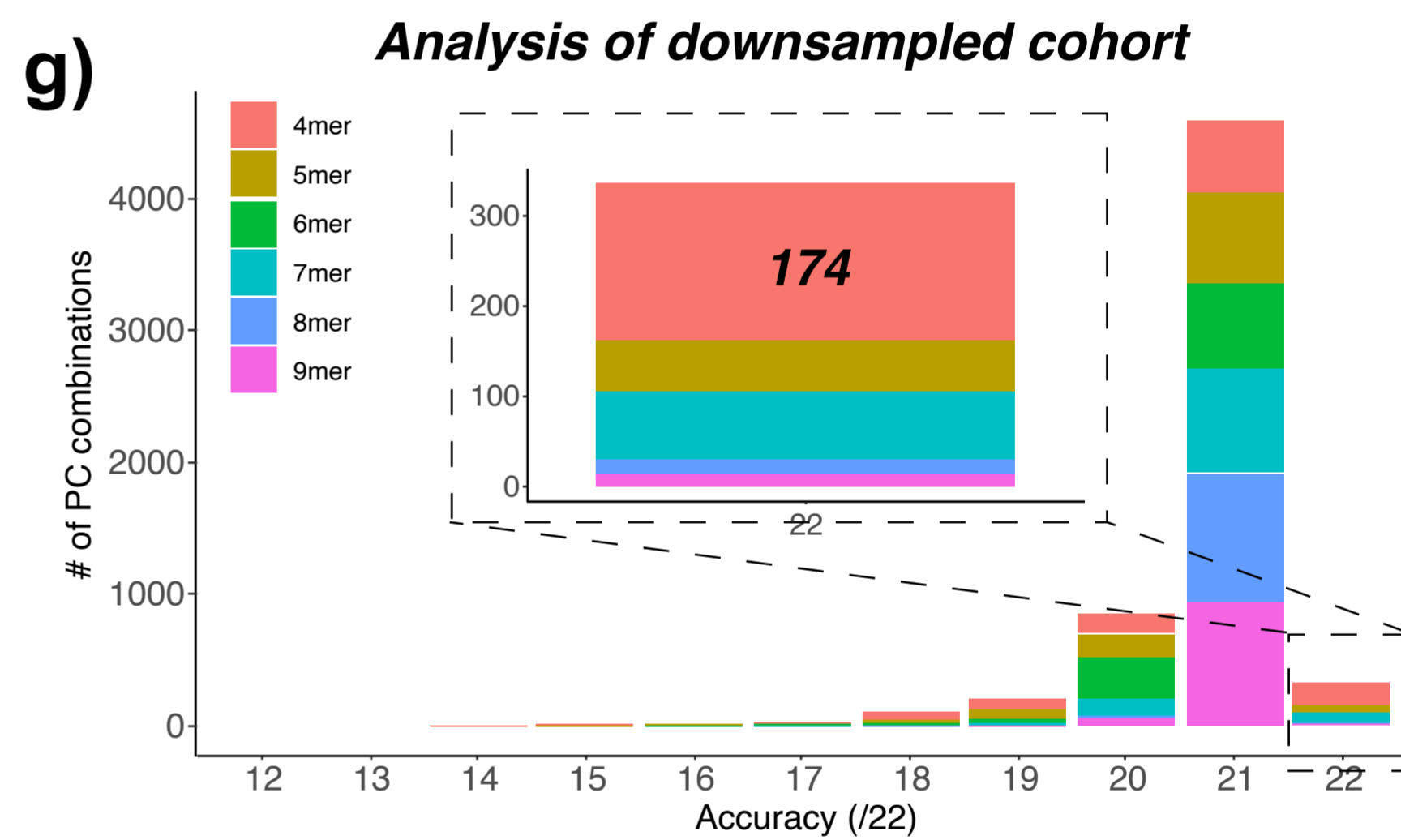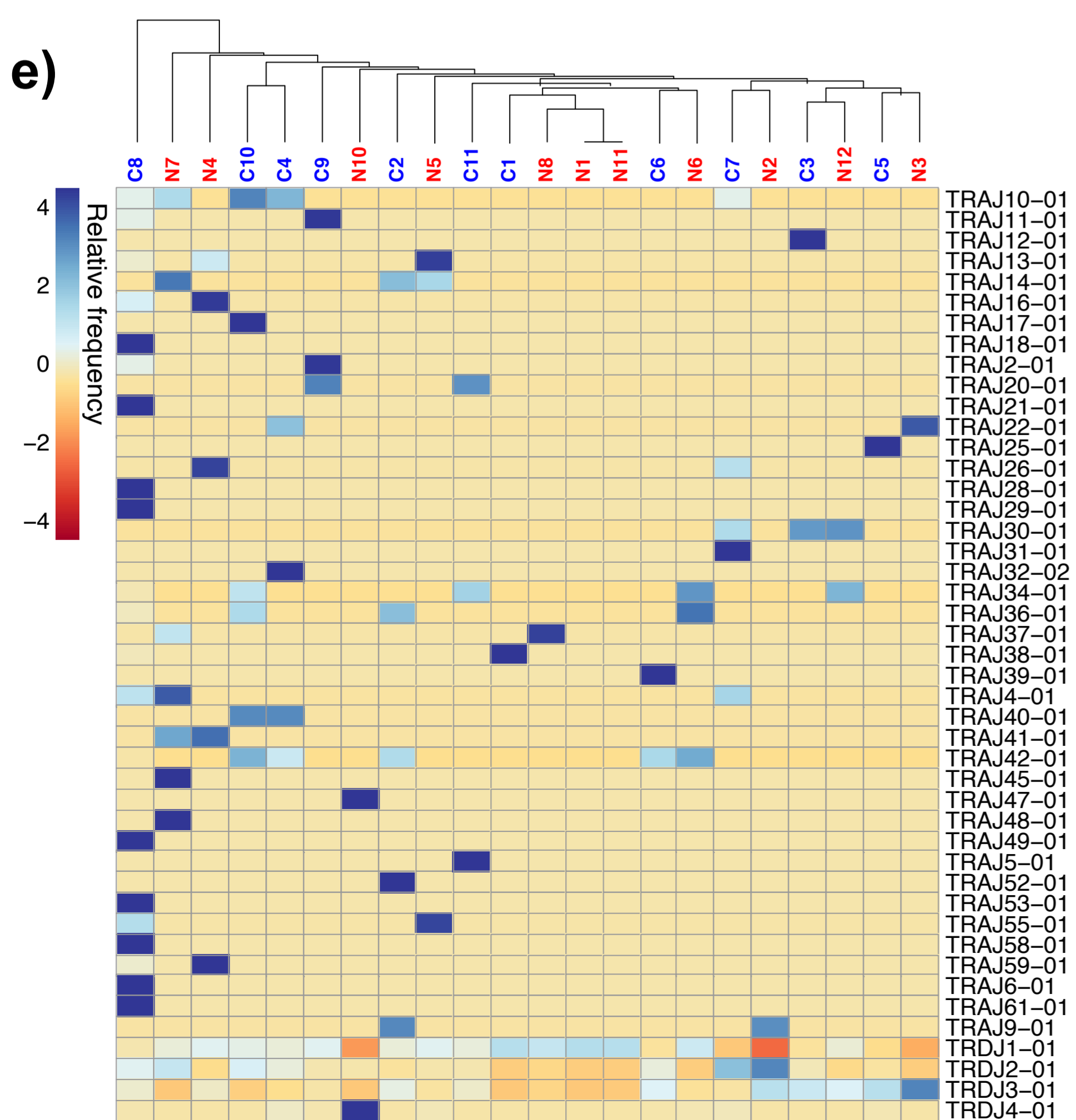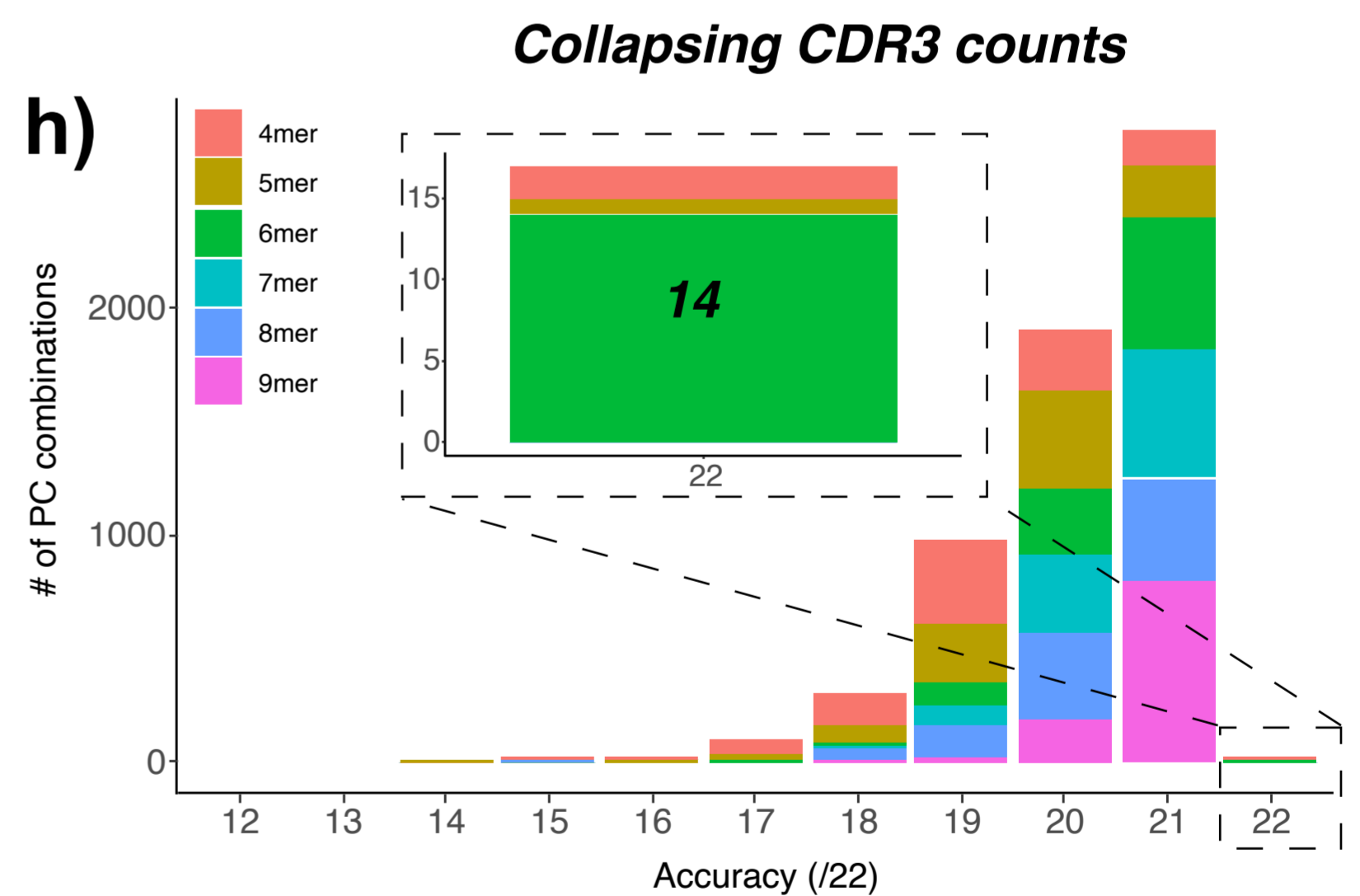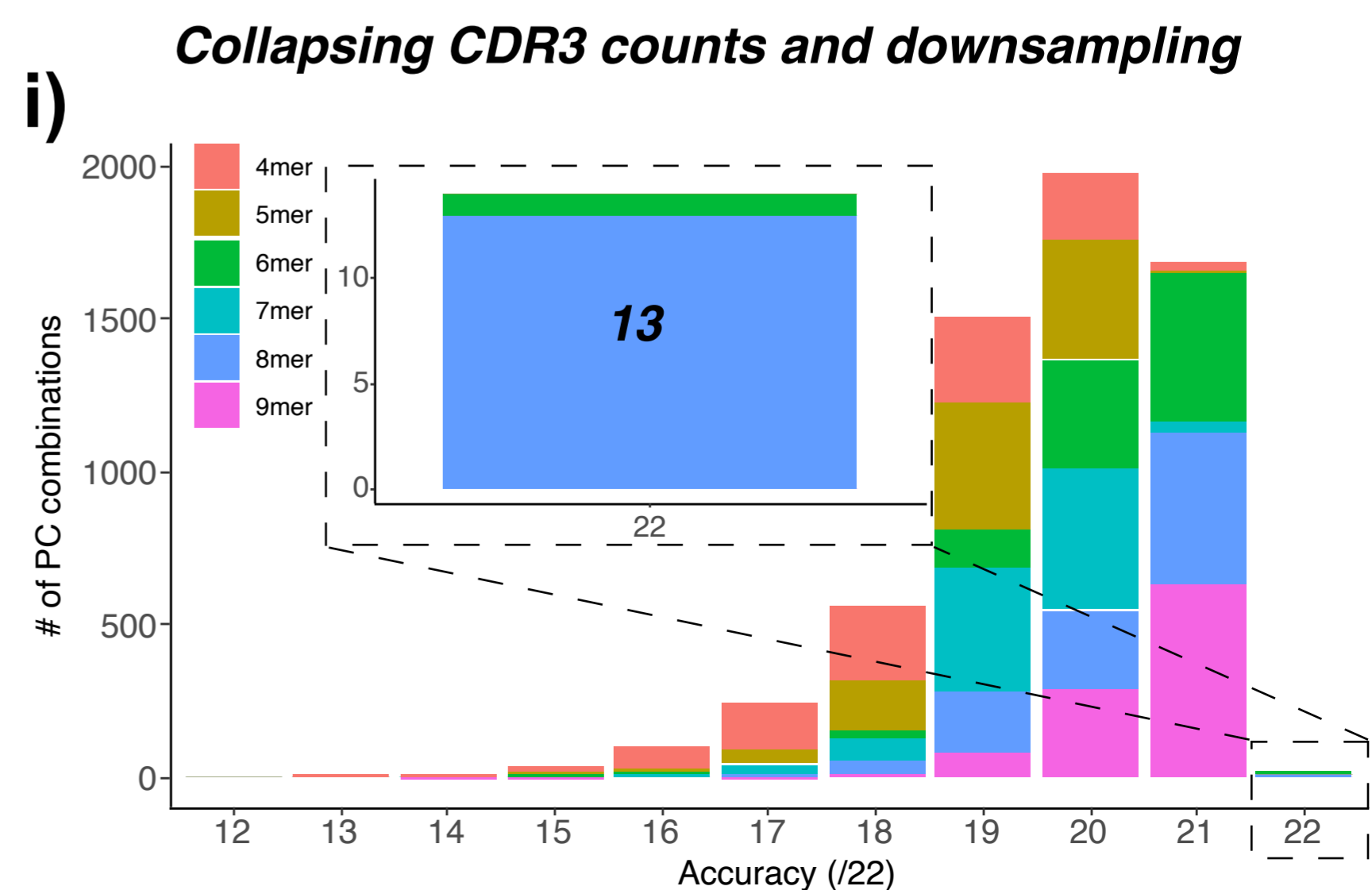

Supplement: Supplementary file 2 — Figure S1. Potentially confounding variables in TRD cannot reliably separate coeliac disease samples from control samples Figure S2. TRD CDR3 length analysis cannot separate coeliac disease samples from control samples Figure S3. Leave‐one‐out cross‐validation for TRD using non‐positional 4mers Figure S4. Potentially confounding variables in TRG cannot reliably separate coeliac disease samples from control samples Figure S5. TRG CDR3 length analysis cannot separate coeliac disease samples from control samples Figure S6. Leave‐one‐out cross‐validation for TRG using positional 5mers Table S1. Details of all study subjects and criteria for inclusion Table S2. Properties of raw and processed TRD and TRG sequence data Table S3. Training accuracy, sensitivity, and specificity results of non‐positional 4mer cluster analysis for TRD Table S4. Training accuracy, sensitivity, and specificity results of positional 7mer cluster analysis for TRD Table S5. Training accuracy, sensitivity, and specificity results of CDR3 cluster analysis for TRD Table S6. Training accuracy, sensitivity, and specificity results of non‐positional 4mer cluster analysis for TRD DNA after random downsampling to the minimum read count Table S7. Training accuracy, sensitivity, and specificity results of non‐positional 4mer cluster analysis for TRD DNA after collapsing of CDR3 sequence data of all the samples in the cohort to a frequency of 1 for every CDR3 sequence Table S8. Training accuracy, sensitivity, and specificity results of non‐positional 4mer cluster analysis for TRD DNA after random downsampling to the minimum read count and collapsing of CDR3 sequence data of all the samples in the cohort to a frequency of 1 for every CDR3 sequence Table S9. Training accuracy, sensitivity, and specificity results of positional 5mer cluster analysis for TRG Table S10. Training accuracy, sensitivity and specificity results of non‐positional 4mer cluster analysis for TRG Table S11. Training accuracy, sensitivity, [file PATH-253-279-s002.zip › path5592-sup-FigureS1.pdf]

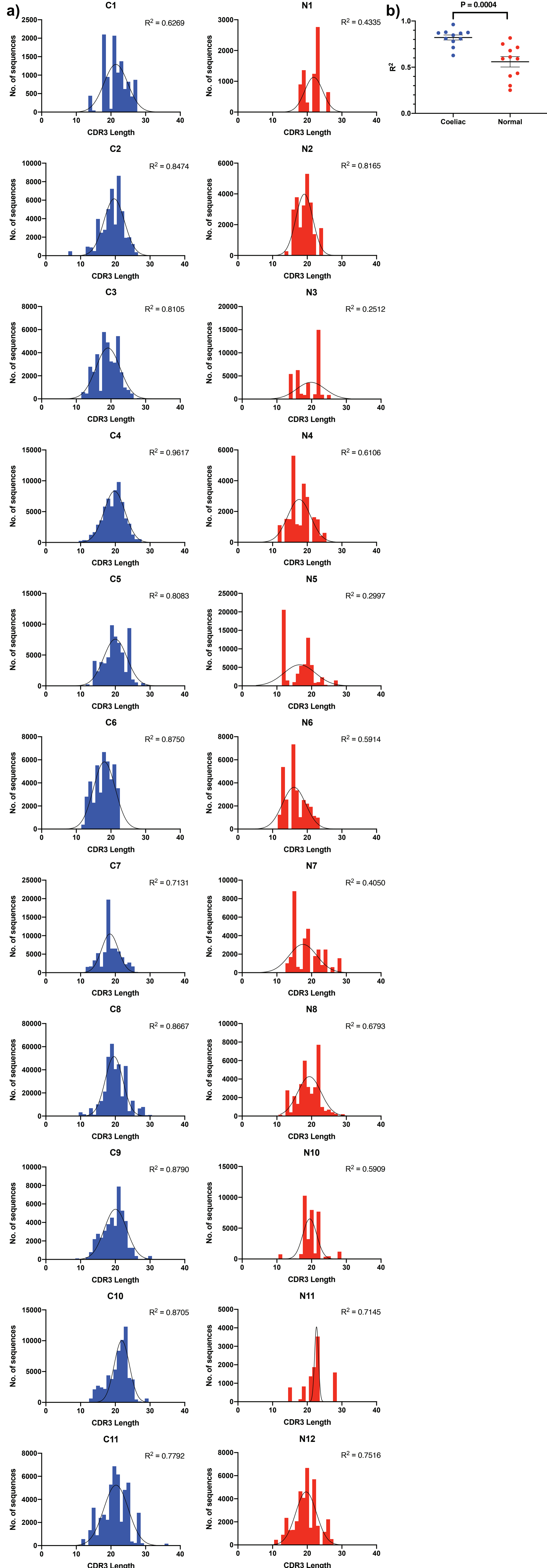

Supplement: Supplementary file 2 — Figure S1. Potentially confounding variables in TRD cannot reliably separate coeliac disease samples from control samples Figure S2. TRD CDR3 length analysis cannot separate coeliac disease samples from control samples Figure S3. Leave‐one‐out cross‐validation for TRD using non‐positional 4mers Figure S4. Potentially confounding variables in TRG cannot reliably separate coeliac disease samples from control samples Figure S5. TRG CDR3 length analysis cannot separate coeliac disease samples from control samples Figure S6. Leave‐one‐out cross‐validation for TRG using positional 5mers Table S1. Details of all study subjects and criteria for inclusion Table S2. Properties of raw and processed TRD and TRG sequence data Table S3. Training accuracy, sensitivity, and specificity results of non‐positional 4mer cluster analysis for TRD Table S4. Training accuracy, sensitivity, and specificity results of positional 7mer cluster analysis for TRD Table S5. Training accuracy, sensitivity, and specificity results of CDR3 cluster analysis for TRD Table S6. Training accuracy, sensitivity, and specificity results of non‐positional 4mer cluster analysis for TRD DNA after random downsampling to the minimum read count Table S7. Training accuracy, sensitivity, and specificity results of non‐positional 4mer cluster analysis for TRD DNA after collapsing of CDR3 sequence data of all the samples in the cohort to a frequency of 1 for every CDR3 sequence Table S8. Training accuracy, sensitivity, and specificity results of non‐positional 4mer cluster analysis for TRD DNA after random downsampling to the minimum read count and collapsing of CDR3 sequence data of all the samples in the cohort to a frequency of 1 for every CDR3 sequence Table S9. Training accuracy, sensitivity, and specificity results of positional 5mer cluster analysis for TRG Table S10. Training accuracy, sensitivity and specificity results of non‐positional 4mer cluster analysis for TRG Table S11. Training accuracy, sensitivity, [file PATH-253-279-s002.zip › path5592-sup-FigureS2.pdf]

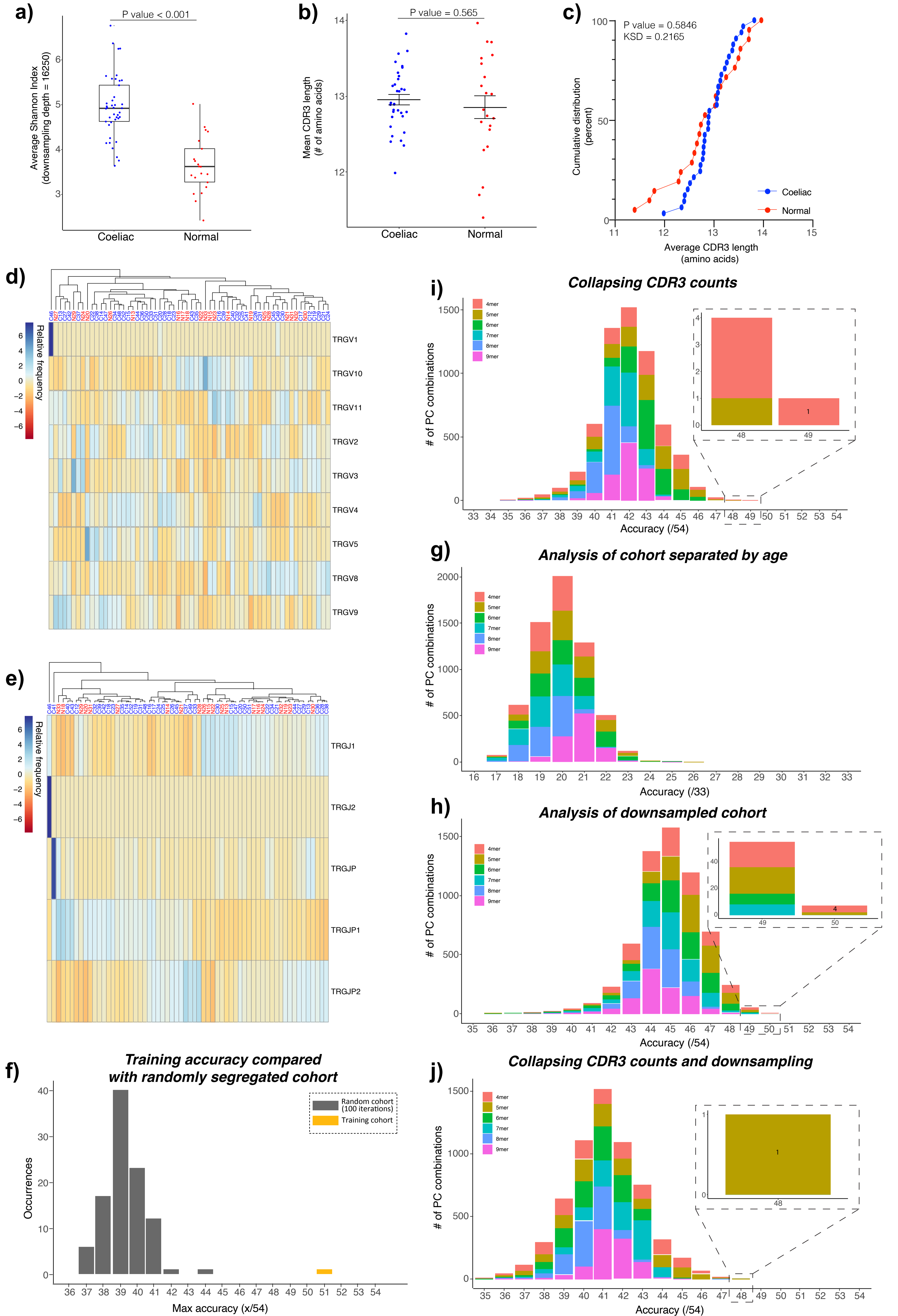

Supplement: Supplementary file 2 — Figure S1. Potentially confounding variables in TRD cannot reliably separate coeliac disease samples from control samples Figure S2. TRD CDR3 length analysis cannot separate coeliac disease samples from control samples Figure S3. Leave‐one‐out cross‐validation for TRD using non‐positional 4mers Figure S4. Potentially confounding variables in TRG cannot reliably separate coeliac disease samples from control samples Figure S5. TRG CDR3 length analysis cannot separate coeliac disease samples from control samples Figure S6. Leave‐one‐out cross‐validation for TRG using positional 5mers Table S1. Details of all study subjects and criteria for inclusion Table S2. Properties of raw and processed TRD and TRG sequence data Table S3. Training accuracy, sensitivity, and specificity results of non‐positional 4mer cluster analysis for TRD Table S4. Training accuracy, sensitivity, and specificity results of positional 7mer cluster analysis for TRD Table S5. Training accuracy, sensitivity, and specificity results of CDR3 cluster analysis for TRD Table S6. Training accuracy, sensitivity, and specificity results of non‐positional 4mer cluster analysis for TRD DNA after random downsampling to the minimum read count Table S7. Training accuracy, sensitivity, and specificity results of non‐positional 4mer cluster analysis for TRD DNA after collapsing of CDR3 sequence data of all the samples in the cohort to a frequency of 1 for every CDR3 sequence Table S8. Training accuracy, sensitivity, and specificity results of non‐positional 4mer cluster analysis for TRD DNA after random downsampling to the minimum read count and collapsing of CDR3 sequence data of all the samples in the cohort to a frequency of 1 for every CDR3 sequence Table S9. Training accuracy, sensitivity, and specificity results of positional 5mer cluster analysis for TRG Table S10. Training accuracy, sensitivity and specificity results of non‐positional 4mer cluster analysis for TRG Table S11. Training accuracy, sensitivity, [file PATH-253-279-s002.zip › path5592-sup-FigureS4.pdf]

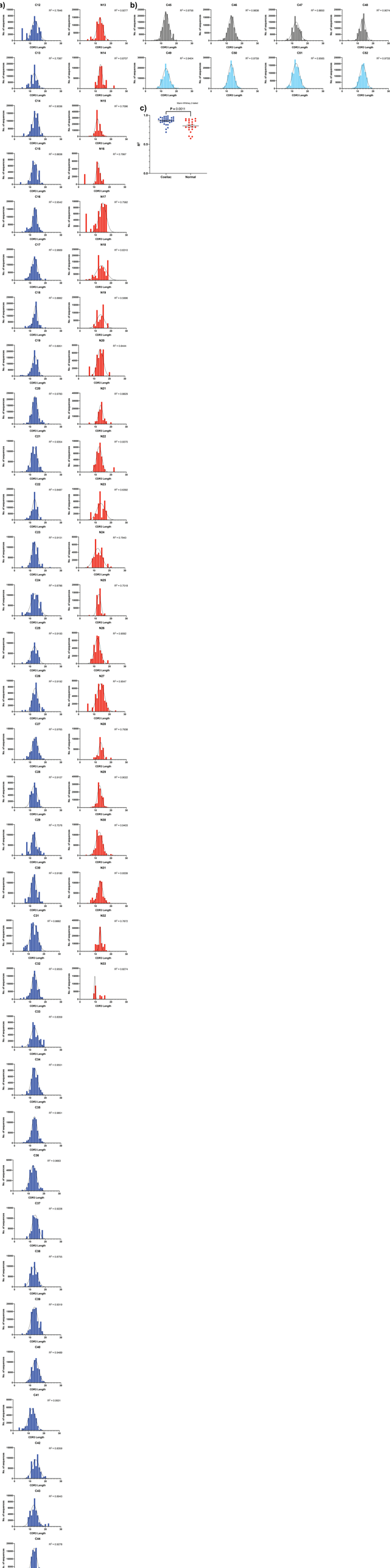

Supplement: Supplementary file 2 — Figure S1. Potentially confounding variables in TRD cannot reliably separate coeliac disease samples from control samples Figure S2. TRD CDR3 length analysis cannot separate coeliac disease samples from control samples Figure S3. Leave‐one‐out cross‐validation for TRD using non‐positional 4mers Figure S4. Potentially confounding variables in TRG cannot reliably separate coeliac disease samples from control samples Figure S5. TRG CDR3 length analysis cannot separate coeliac disease samples from control samples Figure S6. Leave‐one‐out cross‐validation for TRG using positional 5mers Table S1. Details of all study subjects and criteria for inclusion Table S2. Properties of raw and processed TRD and TRG sequence data Table S3. Training accuracy, sensitivity, and specificity results of non‐positional 4mer cluster analysis for TRD Table S4. Training accuracy, sensitivity, and specificity results of positional 7mer cluster analysis for TRD Table S5. Training accuracy, sensitivity, and specificity results of CDR3 cluster analysis for TRD Table S6. Training accuracy, sensitivity, and specificity results of non‐positional 4mer cluster analysis for TRD DNA after random downsampling to the minimum read count Table S7. Training accuracy, sensitivity, and specificity results of non‐positional 4mer cluster analysis for TRD DNA after collapsing of CDR3 sequence data of all the samples in the cohort to a frequency of 1 for every CDR3 sequence Table S8. Training accuracy, sensitivity, and specificity results of non‐positional 4mer cluster analysis for TRD DNA after random downsampling to the minimum read count and collapsing of CDR3 sequence data of all the samples in the cohort to a frequency of 1 for every CDR3 sequence Table S9. Training accuracy, sensitivity, and specificity results of positional 5mer cluster analysis for TRG Table S10. Training accuracy, sensitivity and specificity results of non‐positional 4mer cluster analysis for TRG Table S11. Training accuracy, sensitivity, [file PATH-253-279-s002.zip › path5592-sup-FigureS5.pdf]
